# C/EBPβ/AEP is age-dependently activated in Parkinson’s disease and mediates α-synuclein in the gut and brain

**DOI:** 10.1038/s41531-022-00430-8

**Published:** 2023-01-06

**Authors:** Hualong Wang, Guiqin Chen, Eun Hee Ahn, Yiyuan Xia, Seong Su Kang, Xia Liu, Chang Liu, Ming-Hu Han, Shengdi Chen, Keqiang Ye

**Affiliations:** 1 Department of Neurology and Institute of Neurology, Ruijin Hospital, Shanghai Jiaotong University School of Medicine, Shanghai, 200025 China; 2 Department of Neurology, The First Hospital of Hebei Medical University (Department of Neurology, Hebei Hospital of Xuanwu Hospital Capital Medical University), Brain Aging and Cognitive Neuroscience Laboratory of Hebei Province, Neuromedical Technology Innovation Center of Hebei Province, Shijiazhuang, 050031 Hebei P. R. China; 3 Department of Pathology and Laboratory Medicine, Emory University School of Medicine, Atlanta, Georgia 30322 USA; 4 Department of Neurology, Renmin Hospital of Wuhan University, Wuhan, Hubei Province 430060 China; 5 Department of Physiology, College of Medicine, Hallym University, Hallymdaehak-gil, Chuncheon-si, Gangwon-Do, 24252, South Korea; 6 CAS Key Laboratory of Brain Connectome and Manipulation, The Brain Cognition and Brain Disease Institute (BCBDI), Shenzhen Institute of Advanced Technology, Chinese Academy of Sciences, Shenzhen, Guangdong 518055 China; 7 Shenzhen-Hong Kong Institute of Brain Science-Shenzhen Fundamental Research Institutions, Shenzhen, 518000, China; 8 Department of Mental Health and Public Health, Faculty of Life and Health Sciences, Shenzhen Institute of Advanced Technology, Chinese Academy of Sciences, Shenzhen, Guangdong 518055 China; 9 Department of Biology, Faculty of Life and Health Sciences, Shenzhen Institute of Advanced Technology, Chinese Academy of Sciences, Shenzhen, Guangdong 518055 China

**Keywords:** Parkinson's disease, Cell biology

## Abstract

Parkinson’s disease (PD) is the most common neurodegenerative motor disorder, and its pathologic hallmarks include extensive dopaminergic neuronal degeneration in the Substantia nigra associated with Lewy bodies, predominantly consisting of phosphorylated and truncated α-Synuclein (α-Syn). Asparagine endopeptidase (AEP) cleaves human α-Syn at N103 residue and promotes its aggregation, contributing to PD pathogenesis. However, how AEP mediates Lewy body pathologies during aging and elicits PD onset remains incompletely understood. Knockout of AEP or C/EBPβ from α-SNCA mice, and their chronic rotenone exposure models were used, and the mechanism of α-Syn from the gut that spread to the brain was observed. Here we report that C/EBPβ/AEP pathway, aggravated by oxidative stress, is age-dependently activated and cleaves α-Syn N103 and regulates Lewy body-like pathologies spreading from the gut into the brain in human α-SNCA transgenic mice. Deletion of C/EBPβ or AEP substantially diminished the oxidative stress, neuro-inflammation, and PD pathologies, attenuating motor dysfunctions in aged α-SNCA mice. Noticeably, PD pathologies initiate in the gut and progressively spread into the brain. Chronic gastric exposure to a low dose of rotenone initiates Lewy body-like pathologies in the gut that propagate into the brain in a C/EBPβ/AEP-dependent manner. Hence, our studies demonstrate that C/EBPβ/AEP pathway is critical for mediating Lewy body pathology progression in PD.

## Introduction

Parkinson’s disease (PD), the second most common neurodegenerative disease, is characterized by a progressive loss of nigrostriatal dopaminergic (DAergic) neurons in the substantia nigra pars compacta (SN), thereby inducing a movement disorder. The etiology of PD is multifactorial, implicating both genetic and environmental components. So far, a number of cellular mechanisms, including aberrant protein folding, oxidative stress, and mitochondrial dysfunction have been proposed for the development and progression of this disease. Most cases of PD are sporadic, and their pathogenesis remains unknown. The pathological hallmark of PD contains abnormal α-Synuclein (α-Syn) accumulation in intraneuronal inclusions (Lewy bodies) and neuronal processes (Lewy neurites) along with degeneration of substantia nigra dopaminergic neurons^1^. Autopsy studies indicate an ascending pattern of brain regions in PD pathology^2^. The link between disease progression and increasingly widespread involvement of multiple brain regions is supported by numerous studies demonstrating correlations between neocortical Lewy bodies and dementia in PD^3–5^. Interestingly, Lewy pathology first manifests in the dorsal motor nucleus of the vagus, indicating that this event could result from pathology in the gut that has been propagated via the vagus nerve^6^, although the hypothesis is still under debate^7,8^. The finding of Lewy bodies in the intestinal enteric nerves supports the proposition that the intestine might be an early site of PD-like pathology due to the stimulation by gut luminal -likely bacterial- products, metabolites, or toxins^9^.

Mitochondrial dysfunction and oxidative stress are tightly implicated in the pathogenesis of PD^10–13^. Some forms of familial PD, including PINK1 and DJ1 mutants^14–16^, localize to the mitochondria and gain toxic functions due to the mutations, which elicit mitochondrial dysfunction^17,18^. On the other hand, wild-type and familial mutant forms of α-Syn overexpression in cell cultures and transgenic mouse models trigger cellular changes, including mitochondrial abnormalities^19,20^. The N-terminal 32 amino acids of human α-Syn contain a cryptic mitochondrial targeting signal, essential for mitochondrial targeting of α-Syn^21^. Accumulation of wild-type α-Syn in the mitochondria causes mitochondrial complex I activity to decrease and the production of reactive oxygen species to increase^22^. However, familial α-Syn with A53T mutation incurs these defects at an earlier time point. Importantly, α-Syn lacking a mitochondrial targeting signal failed to aggregate in the mitochondria and showed no detectable effect on complex I function^21^. Administering complex I inhibitors such as rotenone and 1-methyl-4-phenyl-1,2,3,6-tetrahydropyridine (MPTP) to rodents also induces mitochondrial dysfunction, oxidative stress as well as the loss of dopaminergic neurons^23,24^.

Mammalian asparagine endopeptidase (AEP) is an endo-lysosomal cysteine protease that cleaves after asparagine residues. AEP is activated by acidosis during neuro-excitotoxicity and contributes to neuronal loss by degrading DNase inhibitor SET (also known as PHAPII, TAF-Iβ, I2 PP2A), a binding partner of amyloid precursor protein (APP)^25,26^. Moreover, SET inhibits protein phosphatase 2 A (PP2A) and upregulates Tau hyperphosphorylation^27^. We showed that AEP cleaves TDP-43 in the postmortem brain of humans with frontotemporal lobar degeneration (FTLD)^28^. In addition, AEP shreds Tau at N255 and N368 residues, mediating the neurofibrillary tangles (NFT) formation in the human Alzheimer’s disease (AD) brain^29^. Further, AEP cuts APP at N373 and N585 residues, facilitating BACE1 fragmentation of the resultant C-terminal fragment of APP to generate β-amyloid more efficiently. Deletion of AEP alleviates pathological and cognitive defects^29,30^. Furthermore, we showed that AEP cleaved human α-Syn at N103 and enhanced its aggregation and toxicity, induced dopaminergic (DA) neuronal loss and motor disorders, and knockout of AEP diminished α-Syn overexpression-induced dopaminergic loss or motor deficits^31^. Notably, AEP is upregulated by oxidative stress upon MPTP and is implicated in response to inflammation^32,33^. Most recently, we reported that AEP is transcriptionally regulated by C/EBPβ (CCAAT-enhancer-binding protein β), an inflammation-regulated transcription factor. C/EBPβ regulates its transcription and protein levels in an age-dependent manner, which plays a pivotal role in AD pathogenesis via increasing AEP expression^34^. Moreover, C/EBPβ mediates both α-SNCA and MAO-B mRNA transcription, regulating PD pathogenesis^35,36^. In the current study, we show that C/EBPβ/AEP axis is upregulated in an age-dependent manner in a human α-SNCA transgenic mouse model, mediating α-Syn proteolytic cleavage at N103 by active AEP and Lewy body-like pathologies. Consequently, dopaminergic neuronal loss and motor dysfunctions are tightly correlated with these processes. Knockout of C/EBPβ or AEP in these mice diminishes the pathologies and attenuates motor disorders. Strikingly, a chronic low dose of rotenone exposure induces C/EBPβ/AEP signaling activation and Lewy body inclusions in the gut of young α-SNCA mice that spread to the brain. Inactivation of C/EBPβ or AEP abolishes rotenone’s pathological effects.

## Results

### C/EBPβ/AEP axis is activated in human α-SNCA transgenic mice in an age-dependent manner

**Fig. 1:**
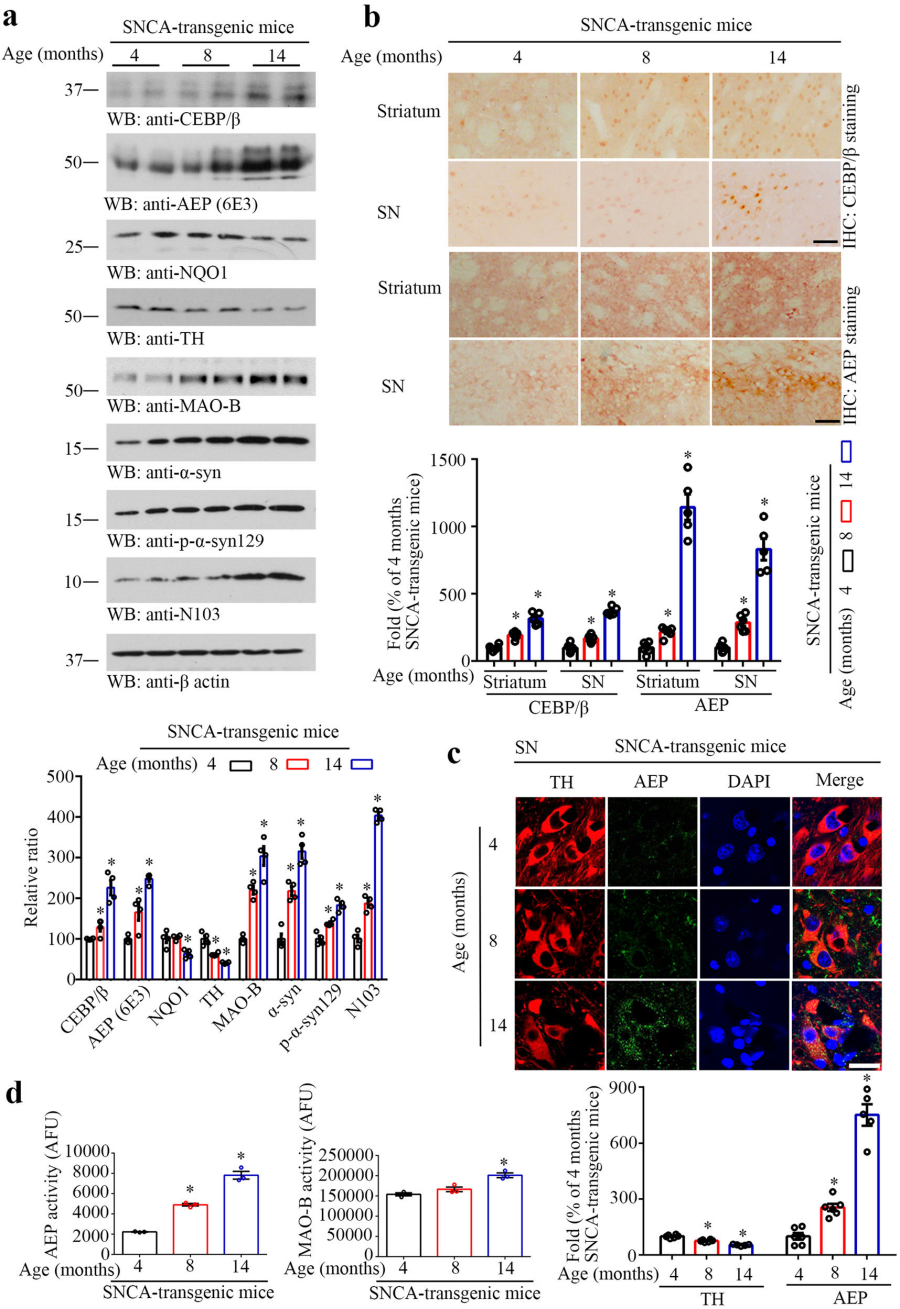
C/EBPβ/AEP axis is age-dependently activated in human α-SNCA transgenic mice. **a** Western blot showing C/EBPβ, AEP, NQO1, TH, MAO-B, and α-Syn expressing and processing in the brain of the α-SNCA transgenic mouse during aging. Quantification of western blotting (*n* = 4 per group, *P < 0.05 *vs* 4 months old α-SNCA transgenic mouse, one-way ANOVA). **b** Immunohistochemistry and the quantification of C/EBPβ and delta-secretase in the striatum and SN (*n* = 5–6 per group, *P < 0.05 vs 4 months old α-SNCA transgenic mouse, one-way ANOVA). Scale bar, 50 μm. Immunofluorescent staining and the quantification (**c**) of TH and AEP in SN (mean ± s.e.m.; *n* = 5–6 per group, *P < 0.05 vs 4 months old α-SNCA transgenic mouse, one-way ANOVA). Scale bar, 20 μm. **d** AEP and MAO-B activity assay in the SN samples (mean ± s.e.m.; *n* = 3 per group, *P < 0.05 vs 4 months old α-SNCA transgenic mouse, one-way ANOVA).

To explore whether C/EBPβ/AEP axis is activated in PD in an age-dependent manner, we monitored expression levels in the brain of human α-SNCA mice at different ages by immunoblotting (Schematic Supplementary Fig. 1a). Both C/EBPβ and AEP were age-dependently escalated, and so were α-Syn and its p-S129 signals. As expected, α-Syn proteolytic truncated product α-Syn N103 and MAO-B levels were also temporally increased. Noticeably, TH levels gradually decreased with age. We made a similar observation with an inducible anti-oxidant enzyme NQO1 (NAD(P)H: quinone oxidoreductase 1), which is neuroprotective to counteract ROS (reactive oxidative species), indicating that ROS are elevated during aging in α-SNCA mice (Fig. 1a). Immunohistochemistry staining (IHC) validated that both C/EBPβ and AEP were age-dependently elevated in both striatum and SN regions (Fig. 1b). Remarkably, immunofluorescent (IF) staining revealed that TH-positive dopaminergic neurons were increasingly lost in the SN, inversely coupled with AEP signals escalation (Fig. 1c). Both AEP and MAO-B activities were steadily increased during aging in α-SNCA mice (Fig. 1d).

Because C/EBPβ and AEP are tightly regulated by oxidative stress and inflammation^32,34,37,38^, we investigated oxidative stress and inflammatory status in human α-SNCA mice. Iba-1 staining revealed that microglia cells were age-dependently activated in both striatum and SN in α-SNCA mice (Supplementary Fig. 1b), and so were the levels of 4-HNE (4-hydroxynonenal), a biomarker for oxidative stress (Supplementary Fig. 1c). In alignment with these observations, pro-inflammatory cytokines were augmented in a similar format (Supplementary Fig. 1d). IHC staining with anti-TH revealed that dopaminergic neuronal loss progressively elevated in both the striatum and SN regions (Supplementary Fig. 1e), fitting with IF staining findings (Fig. 1c). In agreement with these observations, both rotarod and grid behavioral tests demonstrated that α-SNCA exhibits significant motor defects at 14 months of age (Supplementary Fig. 1f), associated with extensive TH loss in both the striatum and SN.

To explore whether Lewy body-like inclusions are temporally augmented in human α-SNCA transgenic mice, we conducted IHC studies and found that AEP-truncated α-Syn N103 escalated in the same way (Supplementary Fig. 2a). Since p-α-Syn S129 is selectively associated with LBs or LNs and colocalizes with ubiquitin (Ub), to confirm that truncated α-Syn are aggregated in the inclusions, we conducted IF co-staining with Thioflavin S (ThS) or Ub and anti-N103. Again, α-Syn N103 and ThS co-staining activities gradually escalated, and so were α-Syn N103 and anti-Ub in the SN regions (Supplementary Fig. 2b, c). Phosphorylated α-Syn was steadily augmented in different brain regions of α-SNCA mice. (Supplementary Fig. 3a). The spatial distribution of p-S129 in the brain at different ages is shown in Supplementary Fig. 3b. Again, p-S129 signals and ThS fluorescent activities were tightly associated with each other and elevated in an age-dependent manner, and so were p-S129 and anti-Ub co-staining activities (Supplementary Fig. 3c, d). Together, our data support that PD pathologies and oxidative stress are escalated in human α-SNCA transgenic mice in an age-dependent manner.

### Depletion of C/EBPβ or AEP diminishes dopaminergic neuronal loss in human α-SNCA transgenic mice

**Fig. 2:**
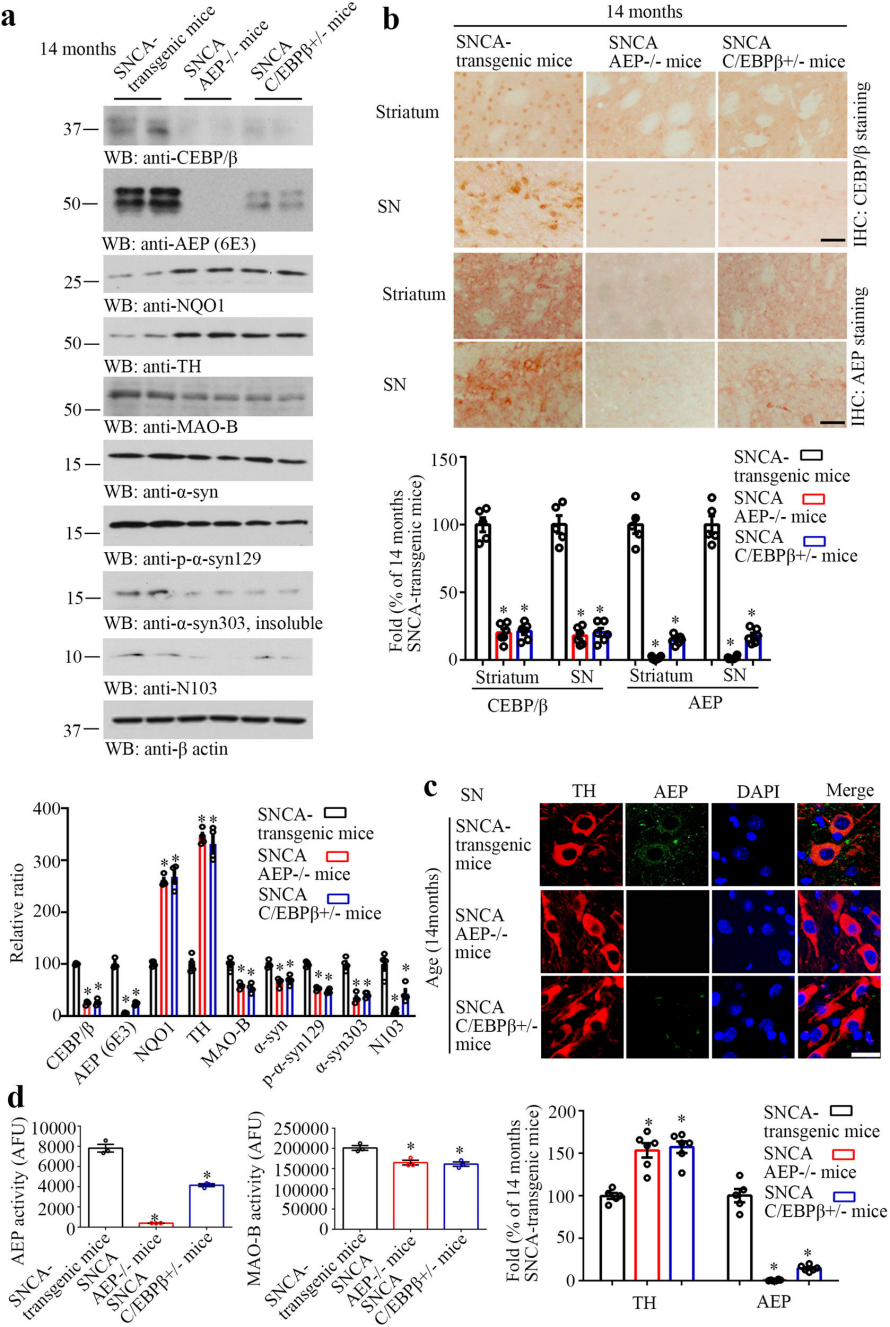
Inactivation of C/EBPβ/AEP pathway rescues dopaminergic neurons in α-SNCA transgenic mice. **a** Western blot showing C/EBPβ, AEP, NQO1, TH, MAO-B, and α-Syn expressing and processing in the brain of α-SNCA transgenic, deletion of AEP and C/EBPβ± from α-SNCA transgenic mice. Quantification of western blotting (*n* = 4 per group, *P < 0.05 vs 14 months old α-SNCA transgenic mouse, one-way ANOVA). **b** Immunohistochemistry and the quantification of C/EBPβ and delta-secretase in the striatum and SN (*n* = 5–6 per group, *P < 0.05 vs 14 months old α-SNCA transgenic mouse, one-way ANOVA). Scale bar, 50 μm. **c** Immunofluorescent staining and the quantification of TH and AEP in SN (mean ± s.e.m.; *n* = 5–6 per group, *P < 0.05 vs 14 months old α-SNCA transgenic mouse, one-way ANOVA). Scale bar, 20 μm. **d** AEP and MAO-B activity assay in the SN samples (mean ± s.e.m.; *n* = 3 per group, *P < 0.05 *vs* 14 months old α-SNCA transgenic mouse, one-way ANOVA).

To assess the pathological roles of the C/EBPβ/AEP axis in PD pathogenesis, we crossed C/EBPβ^+/−^ or AEP^−/−^ with α-SNCA transgenic mice, respectively. Since C/EBPβ^−/−^ displays various defects, including lymphoproliferative disorder, impaired lipid metabolism, live functions, and infertility^39–41^, to avoid potential complications from these defects, we chose C/EBPβ± to breed with α-SNCA mice. Because C/EBPβ is a major transcription factor for AEP, accordingly, AEP was reduced in α-SNCA/C/EBPβ^+/−^ mice. Noticeably, C/EBPβ was barely detectable in α-SNCA/AEP^−/−^ mice, whereas it was demonstrable in 14 months old α-SNCA mice (Fig. 2a, top two panels). Interestingly, deletion of either AEP or C/EBPβ^+/−^ from α-SNCA mice escalated both NQO1 and TH levels as compared to α-SNCA mice. Conversely, both MAO-B and α-Syn levels were reduced, when AEP or C/EBPβ was depleted from α-SNCA mice. Consequently, p-α-Syn S129, α-Syn 303 (insoluble), and N103 levels were robustly decreased compared to α-SNCA mice (Fig. 2a, fourth-bottom panels). AEP elimination and C/EBPβ reduction were validated by IHC staining in both striatum and SN regions (Fig. 2b). IF co-staining showed that TH signals were augmented in α-SNCA/AEP^−/−^ and α-SNCA/C/EBPβ± mice versus α-SNCA mice (Fig. 2c). AEP activities were greatly decreased in C/EBPβ± mice and almost undetectable in AEP^−/−^ mice compared to α-SNCA mice. Moreover, MAO-B enzymatic activities were significantly attenuated in both α-SNCA/AEP^−/−^ and α-SNCA/C/EBPβ^+/−^ mice versus α-SNCA mice (Fig. 2d). IHC staining with anti-N103 demonstrated that α-Syn truncation by AEP was blunted in α-SNCA/AEP^−/−^ mice and suppressed in α-SNCA/C/EBPβ^+/−^ mice (Supplementary Fig. 4a). Both N103/ThS and N103/Ub co-staining were prominently diminished when AEP or C/EBPβ was deleted from α-SNCA mice (Supplementary Fig. 4b, c), and so were the levels of α-Syn mRNA (Supplementary Fig. 4d), suggesting that deletion of AEP or C/EBPβ greatly attenuates the Lewy body-like aggregates in aged α-SNCA mice.

### Inactivation of C/EBPβ or AEP rescues motor impairments in human α-SNCA transgenic mice

**Fig. 3:**
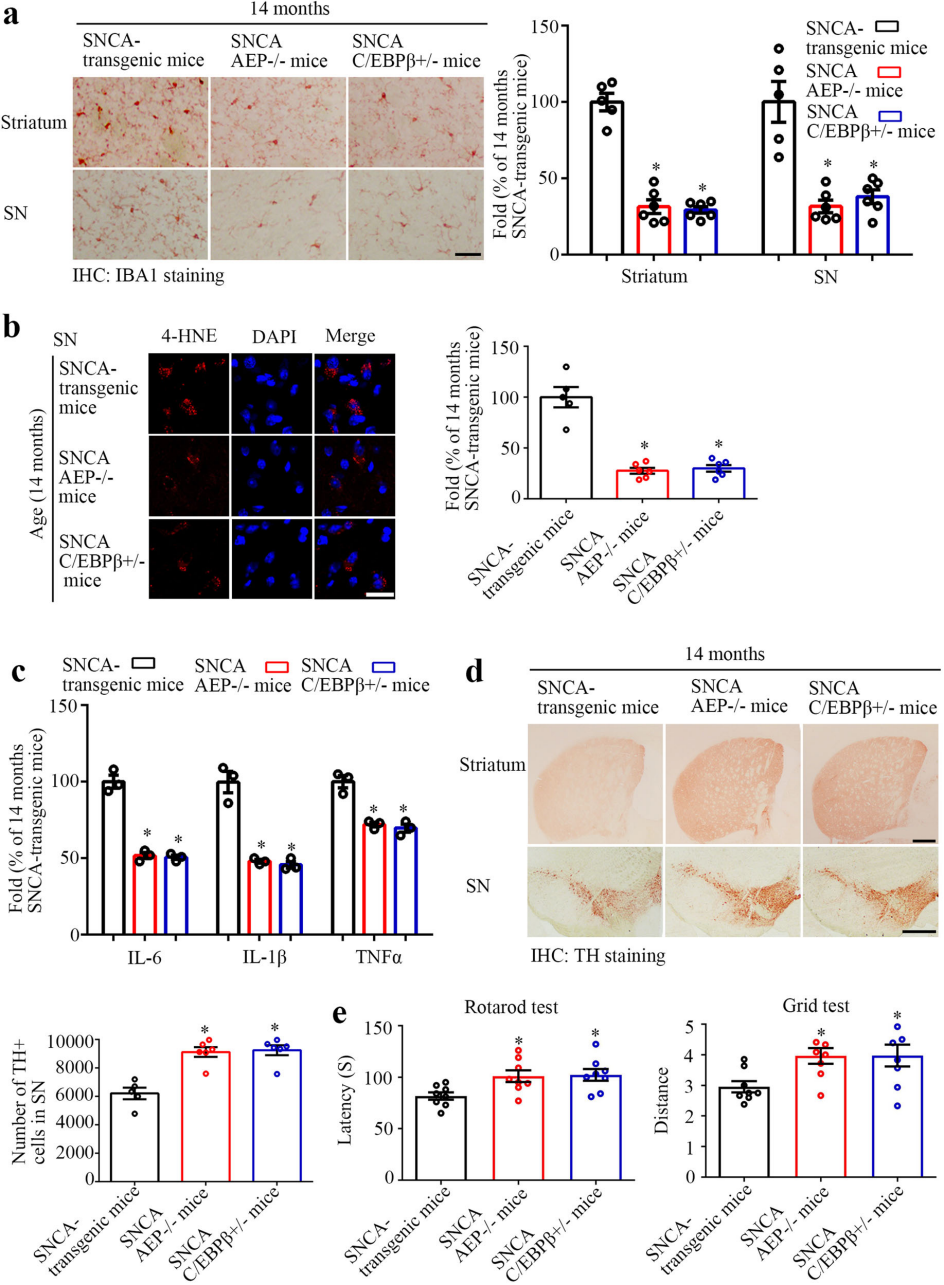
C/EBPβ/AEP pathway mediates neuro-inflammation in α-SNCA transgenic mice. **a** Immunostaining and the quantification of Iba-1 in the striatum and SN. (*n* = 5–6 per group, *P < 0.05 vs 14 months old α-SNCA transgenic mouse, one-way ANOVA). Scale bar, 50 μm. **b** 4-HNE staining and the quantification in the SN. (*n* = 5–6 per group, *P < 0.05 *vs* 14 months old α-SNCA transgenic mouse, one-way ANOVA). Scale bar, 20 μm. **c** Reduction of C/EBPβ and knockdown of delta-secretase decreases the expression levels of inflammatory cytokines in α-SNCA transgenic mice. (*n* = 3 per group, *P < 0.05 vs 14 months old α-SNCA transgenic mouse, one-way ANOVA). **d** Immunostaining of TH in the striatum and SN, and the quantification of TH in SN. (*n* = 5–6 per group, *P < 0.05 vs 14 months old α-SNCA transgenic mouse, one-way ANOVA). Scale bar, 500 μm. **e** Rotarod test and Grid test. (mean ± s.e.m.; *n* = 8 mice per group; *P < 0.05 vs 14 months old α-SNCA transgenic mouse, one-way ANOVA).

To further explore the pathological effects of deleting C/EBPβ or AEP in α-SNCA mice, we performed IHC staining on 14 months old of mice and found that elimination of C/EBPβ or AEP markedly suppressed Iba-1 signals (Fig. 3a). Remarkably, 4-HNE levels were also decreased in α-SNCA/C/EBPβ^+/−^ and α-SNCA/AEP^−/−^ mice as compared to α-SNCA mice (Fig. 3b). Quantitative ROS analysis showed that the oxidative stress was age-dependently escalated in α-SNCA mice, correlated with augmented AEP and Iba-1 signals in the SN regions (Supplementary Fig. 5a, b). Subsequently, inactivation of C/EBPβ or AEP in α-SNCA mice significantly reduced ROS levels, associated with Iba-1 activity decrease in these mice (Supplementary Fig. 5c, d). Consequently, neuro-inflammation was strongly subdued in α-SNCA/C/EBPβ^+/−^ and α-SNCA/AEP^−/−^ mice as compared to α-SNCA mice (Fig. 3c). Anti-TH IHC analysis revealed that knockout of C/EBPβ or AEP significantly increased dopaminergic neurons in the striatum and SN regions of α-SNCA mice (Fig. 3d). In alignment with these observations, motor function behavioral tests showed that both Rotarod and Grid activities were substantially rescued in α-SNCA/C/EBPβ^+/−^ and α-SNCA/AEP^−/−^ as compared to α-SNCA mice (Fig. 3e). Hence, depletion of C/EBPβ or AEP from α-SNCA mice strongly reduces ROS and neuro-inflammation in α-SNCA mice. Inactivation of C/EBPβ or AEP reduces dopaminergic neuronal loss in aged α-SNCA mice, alleviating motor dysfunctions.

### Knockout of C/EBPβ or AEP reduces Lewy body-like aggregations in the gut and the brain

**Fig. 4:**
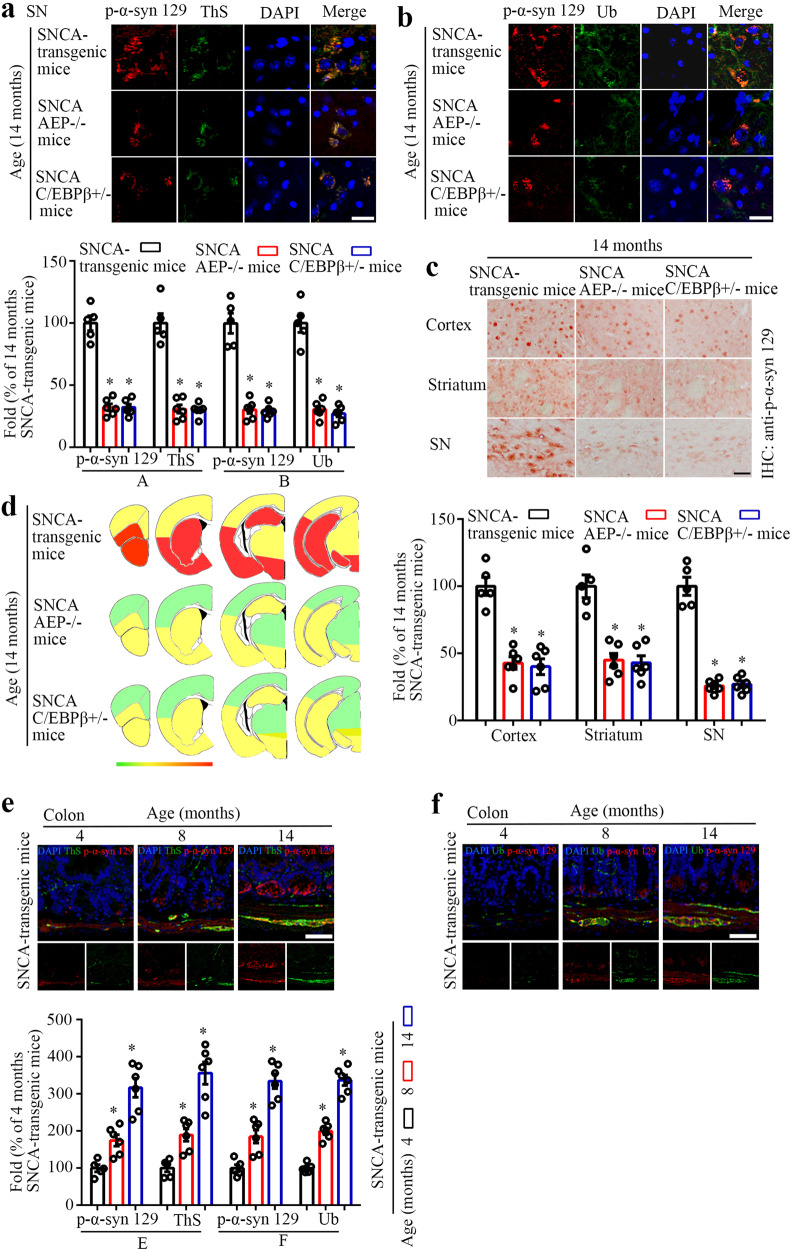
C/EBPβ/AEP signaling regulates Lewy body-like pathology in α-SNCA transgenic mice. Immunofluorescent co-staining of p-α-Syn 129 and ThS (**a**) and p-α-Syn 129 and Ub (**b**) in SN. Quantification of the immunostaining (*n* = 5–6 per group, *P < 0.05 vs 14 months old α-SNCA transgenic mouse, one-way ANOVA). Scale bar, 20 μm. **c** p-α-Syn 129 immunostaining of the cortex, striatum, and SN. Quantification of the IHC staining (*n* = 5–6 per group, *P < 0.05 vs 14 months old α-SNCA transgenic mouse, one-way ANOVA). Scale bar, 50 μm. **d** Heat map colors represent the extent of p-α-Syn 129 pathologies (white (0), no pathology; red (3), the maximal pathology). Immunofluorescent co-staining and the quantification of p-α-Syn 129 and ThS (**e**) and p-α-Syn 129 and Ub (f) in the colon. (*n* = 5–6 per group, *P < 0.05 vs 14 months old α-SNCA transgenic mouse, one-way ANOVA). Scale bar, 50 μm.

To assess whether C/EBPβ or AEP depletion elicited Lewy body-like aggregation reduction in aged α-SNCA mice, we analyzed p-S129 IF co-staining with ThS or Ub in the SN regions. The fluorescent signals of co-staining with p-S129/ThS or p-S129/Ub were attenuated in α-SNCA/C/EBPβ^+/−^ and α-SNCA/AEP^−/−^ as compared to α-SNCA mice (Fig. 4a, b). Moreover, IHC with anti-p-S129 indicated that p-S129 aggregates were reduced in the cortex of both α-SNCA/C/EBPβ^+/−^ and α-SNCA/AEP^−/−^ mice versus α-SNCA mice. Notably, the p-S129 inclusions in SN regions were strongly lessened when C/EBPβ or AEP was depleted from α-SNCA mice (Fig. 4c). The schematic map of the biodistribution of Lewy body-like aggregates was shown (Fig. 4d).

Accumulating evidence supports that Lewy bodies originate from the gut and spread into the brain stem along the vagus nerve^6,42^. To interrogate whether the Lewy body-like inclusions occur in the α-SNCA mice, we performed IHC analysis on the gut sections from different ages of human α-SNCA mice. Both C/EBPβ and AEP staining signals augmented age-dependently in the muscular externa in the colons of α-SNCA mice, correlating with a gradual elevation of AEP-truncated α-Syn N103 (Supplementary Fig. 6a–c, e). Notably, p-S129 activities were strongly increased in both mucosa and muscularis externa in the colon 14 months old of α-SNCA mice, fitting with augmented α-Syn N103 activities (Supplementary Fig. 6d, e). As well, p-S129 was mainly expressed in the TH-positive cells (Supplementary Fig. 6f). IF co-staining on the gut tissues also indicated that both p-S129/ThS and p-S129/Ub signals temporally escalated. Thus, these findings indicate that the Lewy body-like aggregates are increased during aging in the colon of α-SNCA mice (Fig. 4e, f). Similar to what occurred in the brain, depletion of C/EBPβ or AEP from α-SNCA mice greatly blunted α-Syn N103 production (Supplementary Fig. 7a–c, e). Consequently, p-S129 activities were also robustly diminished in α-SNCA/C/EBPβ^+/−^ and α-SNCA/AEP^−/−^ mice versus α-SNCA mice (Supplementary Fig. 7d, e). IF co-staining with p-S129 and ThS or Ub confirmed that Lewy body-like inclusions were pronouncedly mitigated in the colon of α-SNCA mice when either C/EBPβ or AEP was depleted (Supplementary Fig. 7f, g). Therefore, Lewy body-like pathologies in the gut of α-SNCA mice are also mediated by C/EBPβ/AEP axis.

### Chronic gastric Rotenone exposure elicits C/EBPβ/AEP signaling activation, constipation, and PD pathologies in young α-SNCA mice

**Fig. 5:**
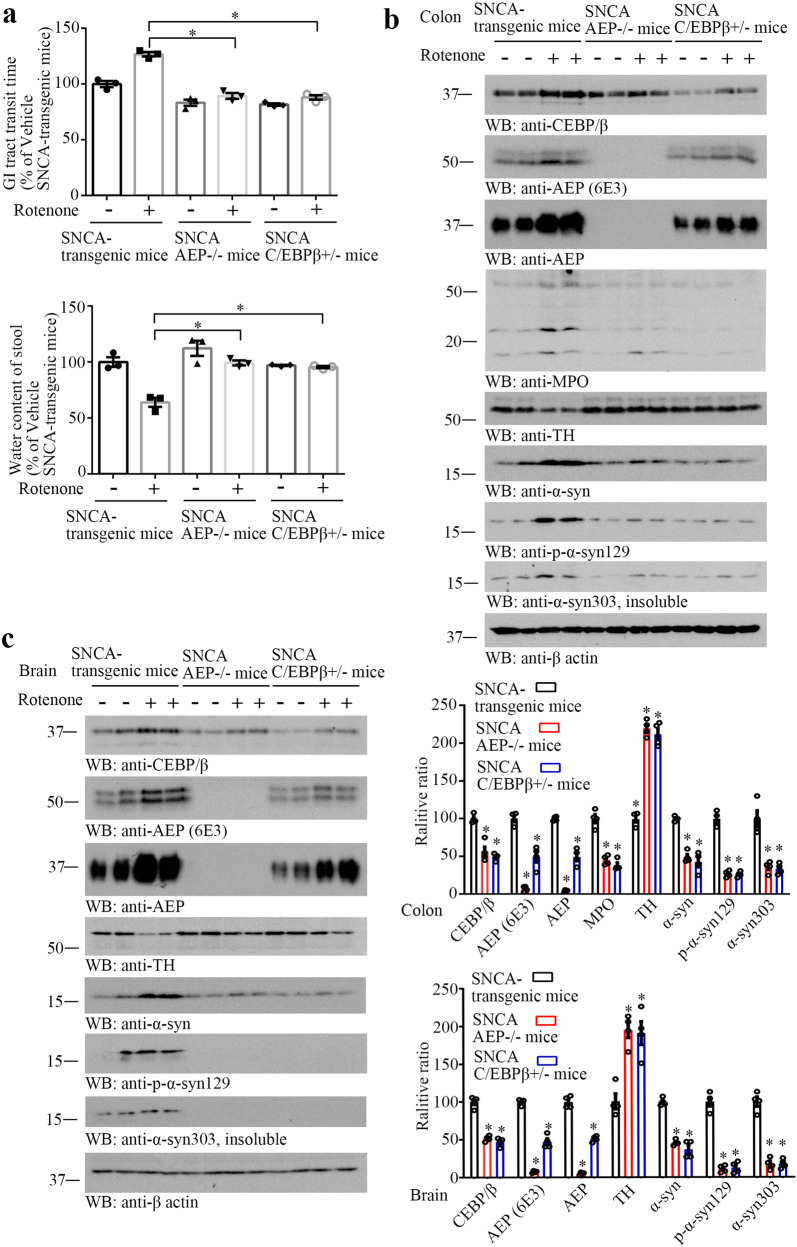
Knockout of C/EBPβ/AEP pathway alleviates constipation in α-SNCA transgenic mice. **a** Gastrointestinal (GI) tract transit time and water content of stool in the mice. (*n* = 3 per group, *P < 0.05 vs rotenone treated α-SNCA transgenic mouse, one-way ANOVA). Western blot showing C/EBPβ, AEP, MPO, TH, α-Syn, and p-α-Syn 129 expressing in the mouse colon (**b**) and C/EBPβ, AEP, TH, α-Syn, and p-α-Syn 129 expressed in the mouse brain (**c**). Quantification of western blotting (*n* = 4 per group, *P < 0.05 *vs* rotenone treated α-SNCA transgenic mouse, one-way ANOVA).

Our data indicate that C/EBPβ is barely detectable in α-SNCA mice, when AEP is knocked out, suggesting that AEP might feedback and modulate C/EBPβ expression as well. To investigate whether C/EBPβ/AEP axis plays an essential role in the gut pathologies associated with PD, we treated young α-SNCA, α-SNCA/AEP^−/−^ and α-SNCA/C/EBPβ^+/−^ mice (1-month-old) with 2.5 mg/kg of rotenone or vehicle control (P.O., once a day) consecutively for 3 months. Gut motility and stool moisture showed that chronic rotenone but not vehicle exposure triggered severe constipation in α-SNCA mice; in contrast, these effects were abolished in α-SNCA/AEP^−/−^ and α-SNCA/C/EBPβ^+/−^ mice, indicating that C/EBPβ/AEP somehow mediates these prodromal effects in PD (Fig. 5a). Immunoblotting with the colon tissues showed that rotenone promoted C/EBPβ expression that resulted in AEP upregulation in α-SNCA mice. Accordingly, TH levels were prominently reduced, inversely coupled with α-Syn, p-α-Syn S129, and α-Syn 303 (insoluble) elevation. By contrast, these effects were pronouncedly attenuated in both α-SNCA/AEP^−/−^ and α-SNCA/C/EBPβ^+/−^ mice (Fig. 5b). We made similar observations in the SN regions of the brain of these animals (Fig. 5c). Quantification analysis also supported that TNFα, IL-1β, and IL-6 inflammatory cytokines were strongly augmented in the brain and gut tissues of α-SNCA by rotenone compared to vehicle, and these effects were robustly suppressed in α-SNCA/AEP^−/−^ and α-SNCA/C/EBPβ^+/−^ mice (Supplementary Fig. 8a, b). AEP was robustly activated by rotenone in both the SN and colon of α-SNCA mice, which was diminished in α-SNCA/AEP^−/−^ and α-SNCA/C/EBPβ^+/−^ mice (Supplementary Fig. 8c). MPO (myeloperoxidase), a biomarker for inflamed colon^43^, was strongly increased and fragmented in α-SNCA but not α-SNCA/AEP^−/−^ mice upon rotenone, suggesting that the gut is harshly inflammatory (Fig. 5b, fourth panel). Hence, chronic rotenone exposure incurred TH-positive-positive dopaminergic neuronal degeneration in the SN and colon of α-SNCA mice, which is mediated by the C/EBPβ/AEP pathway. Therefore, chronic rotenone initiates C/EBPβ/AEP axis activation in the gut and induces constipation and gut pathologies in α-SNCA mice in an AEP-dependent manner.

### Rotenone triggers α-Syn aggregates propagation from the gut into the brain in an AEP-dependent way

**Fig. 6:**
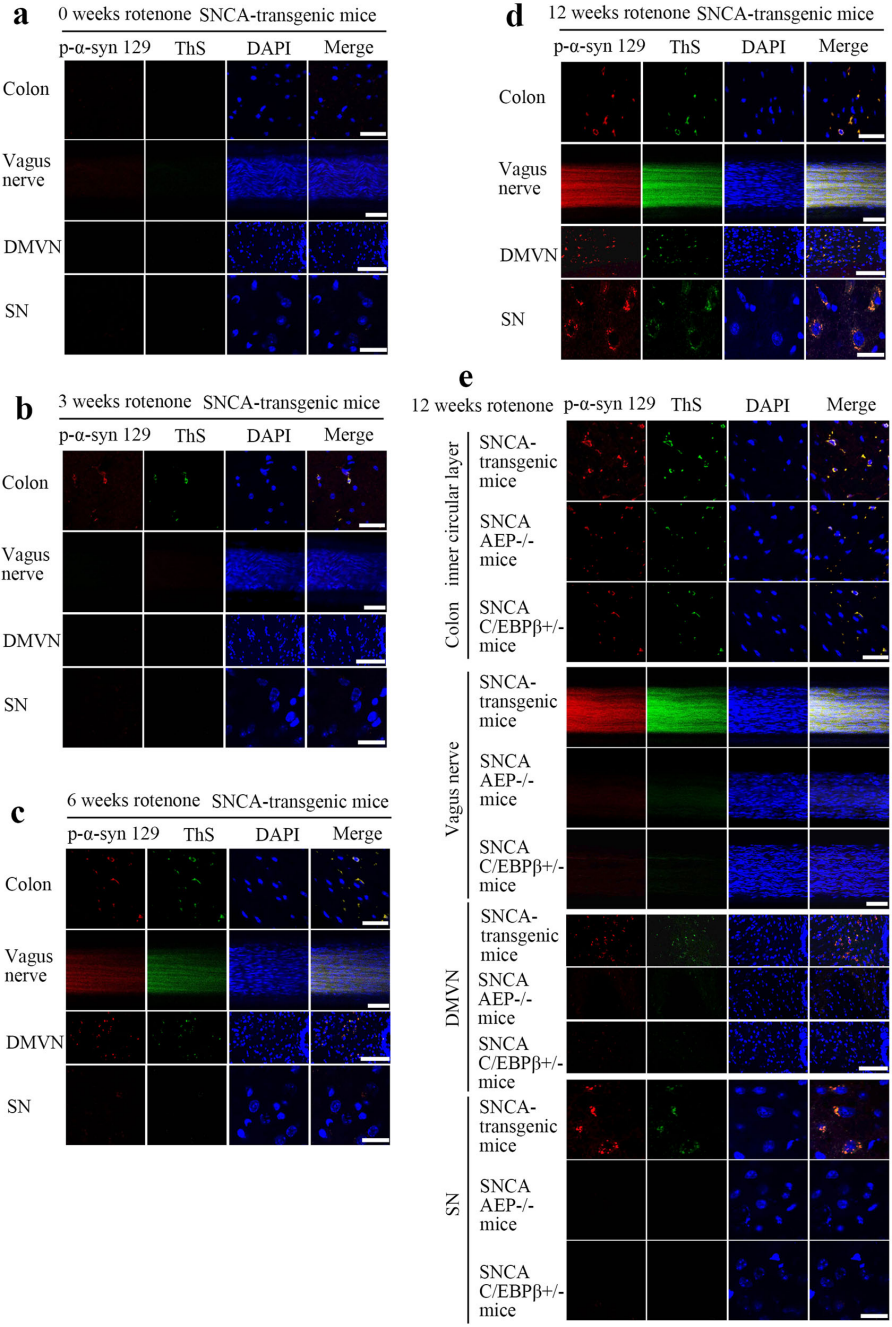
C/EBPβ/AEP axis mediates Lewy body-like pathology propagation from the gut into the brain in α-SNCA mice. Immunofluorescent co-staining of p-α-Syn 129 and ThS in the colon (Scale bar, 20 μm), vagus nerve (Scale bar, 50 μm), DMVN (Scale bar, 100 μm), and SN (Scale bar, 20 μm) in the α-SNCA transgenic mouse after 0-week (**a**), 3 weeks (**b**), 6 weeks (**c**), and 12 weeks (**d**) rotenone treatment. **e** Reduction of C/EBPβ and knockdown of AEP block the spreading of p-α-Syn 129 co-stained with ThS.

Previous studies demonstrate that rotenone-induced PD pathologies in the brain are mediated by the vagus nerve^44^. Moreover, our most recent study shows that α-Syn N103/Tau N368 fibrils, inoculated in the muscular layer of the colon, spread into the brain stem along the vagus nerve. Moreover, resection of the vagus nerve diminishes the fibrils propagation and inhibits fibrils-induced PD pathologies in the brain^45^. To explore whether the rotenone-induced C/EBPβ/AEP axis mediates the spreading of α-Syn aggregates in the colon into the brain via the vagus nerve, we treated α-SCNA mice for different time points and monitored α-Syn inclusions temporal translocation in the colon, vagus nerve, brain stem (DMVN) and SN. IF staining revealed that Lewy body-like pathology that was both p-α-Syn S129/ThS positive were detectable in the colon 3 weeks after rotenone treatment, which propagated along the vagus nerve and reached DMVN at 6 weeks. The Lewy body-like pathology propagated into the SN 12 weeks after rotenone stimulation (Fig. 6a–d). By contrast, the Lewy body-like pathology spreading events triggered by rotenone were abolished in both α-SNCA/AEP^−/−^ and α-SNCA/C/EBPβ^+/−^ mice (Fig. 6e). Thus, our findings support that C/EBPβ/AEP axis plays a critical role in mediating Lewy body propagation and PD progression.

### C/EBPβ/AEP pathway correlates with Lewy body pathologies in human PD patients

**Fig. 7:**
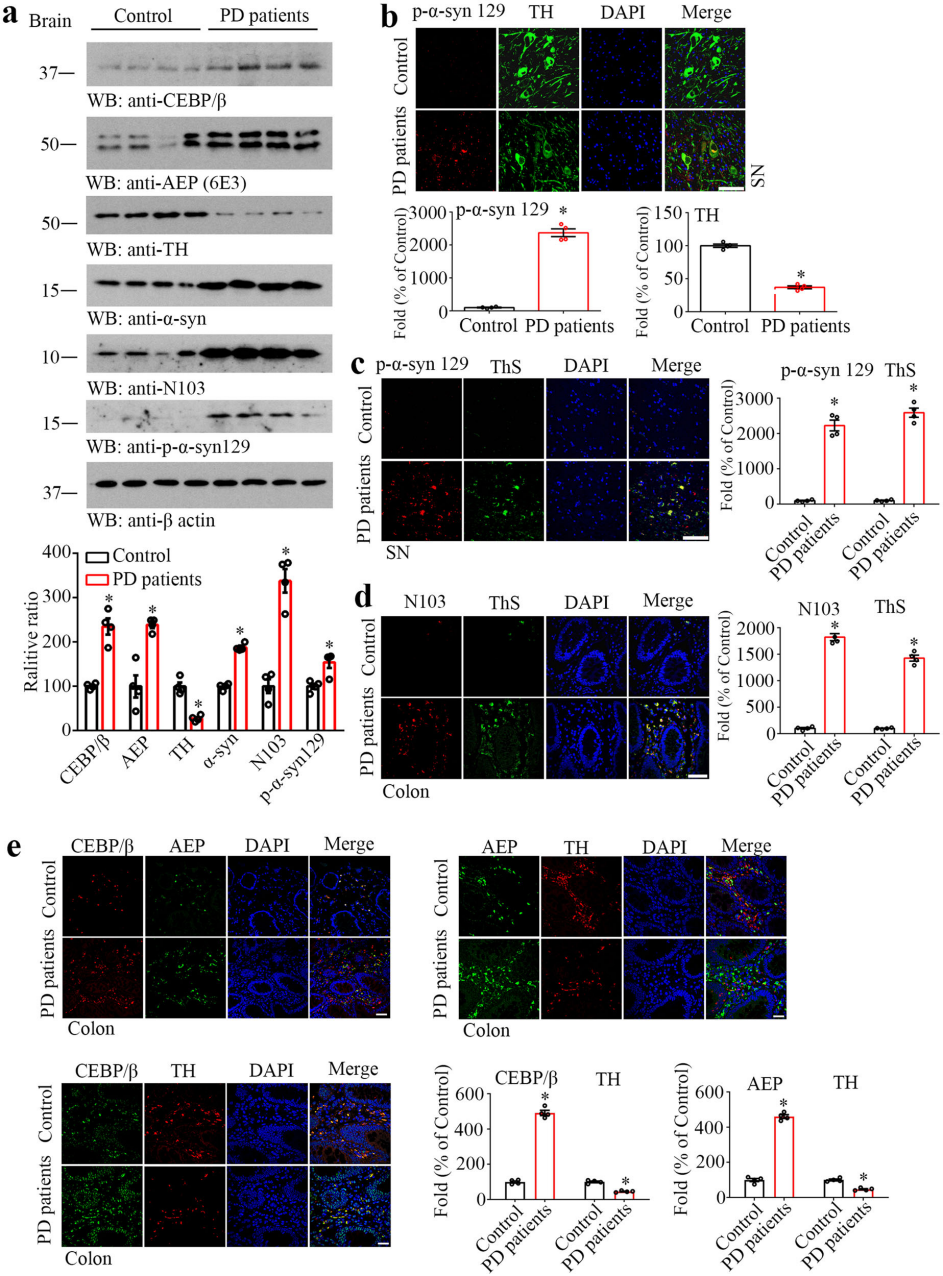
C/EBPβ/AEP signaling is upregulated in PD patient brain and gut. **a** Western blot and the quantification showing C/EBPβ, AEP, TH, and α-Syn expressing and processing in PD patients and Control. (*n* = 4 per group, *P < 0.05 vs Control, Student’s *t*-test, two-tailed). Immunofluorescent co-staining of p-α-Syn 129 and TH (**b**) and co-staining of p-α-Syn 129 and ThS (**c**) in SN of PD patients and Control. Quantification of the immunostaining (*n* = 4 per group, *P < 0.05 vs Control, Student’s *t*-test, two-tailed). Scale bar, 100 μm. Immunofluorescent co-staining of N103 and ThS (**d**) and co-staining of CEBP/β and AEP, CEBP/β and TH, AEP, and TH (**e**) in the colon of PD patients and healthy control (human biopsy samples with the mucosal/submucosal layer). Quantification of the immunostaining (*n* = 4 per group, *P < 0.05 vs Control, Student’s *t*-test, two-tailed). Scale bar, 50 μm.

To investigate whether C/EBPβ/AEP signaling pathway is also implicated in human PD patients, we conducted immunoblotting with PD brain samples. Both C/EBPβ and AEP were escalated in PD brains compared with health control, associated with a prominent increase of both α-Syn and AEP-specific truncated N103 signals. Accordingly, p-α-Syn S129 levels were highly elevated in PD brains, inversely coupled with robust TH reduction (Fig. 7a). IF staining on brain sections with antibodies against both p-α-Syn S129 and TH also validated these findings (Fig. 7b). p-α-Syn S129 and ThS co-staining revealed abundant Lewy bodies in PD brains but not in control (Fig. 7c). IF analysis on PD colon sections also demonstrated extensive Lewy bodies co-stained with anti-α-Syn N103, indicating that AEP was strongly activated in PD patient gut as compared to healthy controls (Fig. 7d). Furthermore, C/EBPβ was highly upregulated in PD patient colon tissues, coupled with robust AEP signals, which were expressed in TH-positive cells, in comparison to those in healthy controls (Fig. 7e). Together, our studies support that C/EBPβ/AEP axis plays an essential role in regulating PD pathologies onset and progression.

## Discussion

In the current report, we show that C/EBPβ/AEP axis is age-dependently upregulated and activated in α-SNCA transgenic mice and PD patients. Moreover, ROS and neuro-inflammation were temporally escalated in α-SNCA mice, fitting with C/EBPβ and AEP augmentation because both of them are strongly regulated by oxidative stress and inflammation^34,38^. In alignment with these findings, NQO1, an inducible antioxidative enzyme, was temporally reduced in the α-SNCA mice. Further, C/EBPβ/AEP signaling activation correlates with α-Syn cleavage by active AEP at N103, correlating with α-Syn aggregation and Lewy body-like inclusions in both the brain and the colon from α-SNCA mice. As expected, dopaminergic neurons were extensively lost in aged α-SNCA mice, associated with significant motor impairments. Remarkably, knockout of AEP or C/EBPβ from α-SNCA mice strongly decreases neuro-inflammation and ROS and PD pathologies, alleviating motor dysfunctions. Thus, these findings support that C/EBPβ/AEP signaling plays a critical role in mediating PD pathogenesis, presumably via cleaving human α-SNCA into N103 fragment and promoting its aggregation. It is worth noting that C/EBPβ/AEP axis is activated in the ENS (enteric neuronal system) and mediates α-Syn aggregation in the gut. Chronic rotenone exposure elicits C/EBPβ activation that leads to AEP upregulation, which subsequently cleaves α-Syn at N103 in enteric neurons. The truncated α-Syn N103 is prone to aggregate^31^ and forms fibrillary inclusions in the gut that spread to the brain via the vagus nerve (Fig. 6a–d).

ROS mainly results from mitochondria, as it utilizes oxygen for energy production. ROS and RNS are normally generated by tightly regulated enzymes. For instance, excessive stimulation of NAD(P)H and electron transport chain triggers the overproduction of ROS, resulting in oxidative stress^46^. The interaction of α-Syn with mitochondria elicits the release of cytochrome c, an increase of mitochondrial calcium and nitric oxide, and oxidative modification of mitochondrial components, suggesting that mitochondria play a pivotal role in oxidative stress and apoptosis induced by α-Syn^22^. On the other hand, Rotenone and 1-methyl-4-phenylpyridinium (MPP+) share a common mode of action as specific inhibitors of mitochondrial complex I^47^, its deficiency is a major factor in neurodegeneration^23^ and parkinsonian symptoms are observed in drug (i.e., MPTP) addicts^48^. Hence, MPTP is broadly employed to induce parkinsonian pathology in animal models. Mitochondria are vulnerable to oxidative stress-induced damage with a number of key biomolecules being the target of oxidative damage by free radicals, including membrane phospholipids, respiratory chain complexes, proteins, and mitochondrial DNA (mt DNA). Consequently, a deficit in cellular energy status may occur along with increased electron leakage and partial reduction of oxygen, which, in turn, leads to a further ROS production escalation. Oxidative damage to certain mitochondrial biomolecules is implicated in the pathophysiology of a number of diseases^49,50^. Thus, excessive oxidative stress might be the main cause of the etiology of many diseases, including PD and AD.

NQO1 is regulated by Nrf2^51^, and its plays an important role in the metabolites of dopamine oxidation-induced oxidative stress^52^. The cellular changes of NQO1 in substantia nigra stand for one of the biochemical characteristics of PD^53^. NQO1 levels are increased in the early and intermediate stages of PD and disappear at the end stage of the disease^52^. Interestingly, sulforaphane induces NQO1 and protects against neuro-cytotoxicity associated with dopamine quinone in vitro^54^ and against MPTP-elicited toxicity in vivo^55^. Accordingly, ROS levels escalate during aging, suppressing NQO1 expression (Fig. 1a). C/EBPα regulates NQO2, a homolog of NQO1 transcription^56^. Conceivably, C/EBPβ might mediate NQO1 mRNA transcription, mediating the ROS levels in PD pathologies. Dopamine-derived ROS and oxidized dopamine metabolites are toxic to DAnergic neurons^57^. However, neuro-inflammation also play a key role in PD etiology. Activated microglia and increased levels of inflammatory mediators are detected in the striatum of deceased PD patients, and numerous animal studies support a contributory role of inflammation in dopaminergic cell loss^58,59^. In alignment with these observations, we found that neuro-inflammation is elevated in an age-dependent manner in α-SNCA transgenic mice (Supplementary Fig. 1a, c). Postmortem human subjects also revealed the presence of activated microglia decades after MPTP exposure, suggesting that even a brief pathogenic insult can induce an ongoing inflammatory response^60^. Activated glia affects neuronal injury and death via generating neurotoxic factors like tumor necrosis factor-alpha (TNFα), prostaglandins, and reactive oxygen and nitrogen species. As the disease continues, inflammatory secretions engage neighboring cells, such as astrocytes and endothelial cells, triggering a vicious cycle of autocrine and paracrine inflammation elevation resulting in neurodegeneration^61^. C/EBPβ regulates pro-inflammatory gene expression in glial activation^62^. Consequently, the deletion of C/EBPβ strongly suppresses the inflammation in α-SNCA mice (Fig. 3a, c). Noteworthily, C/EBPβ expression is almost undetectable in α-SNCA/AEP^−/−^ mice, though it is demonstrable in α-SNCA mice (Fig. 2a). This finding indicates that not only C/EBPβ could act as a transcription factor for AEP, and AEP feeds back and mediates C/EBPβ expression as well^34^. The results also show that α-Syn mRNA and its expression levels are reduced in C/EBPβ or AEP knockout mice (Supplementary Fig. 4d). Our previous study has suggested that C/EBPβ acts as a transcription factor for α-Syn^36^, supporting that C/EBPβ and AEP modulate the α-Syn mRNA transcription and its protein expression.

C/EBPβ is involved in the regulation of pro-inflammatory gene expression in glial activation and plays a key role in the induction of neurotoxic effects by activated microglia^62^. We also observed an age-dependent microglia activation in α-SNCA mice, for which the C/EBPβ/AEP pathway is indispensable because depletion of either of them substantially represses Iba-1 activation in α-SNCA mice (Fig. 3a). Interestingly, dopamine dose-dependently induces C/EBPβ expression in human neuroblastoma cells, associated with temporal escalation of α-Syn, suggesting that C/EBPβ might be implicated in PD pathologies^63^. Most recently, we show that OGD (oxygen-glucose deprivation) upregulates AEP via activating C/EBPβ, which acts as a major transcription factor for AEP and regulates its mRNA expression in aging and AD brain^34^. In some of the cells in the gut, where both of them are colocalized and C/EBPβ may mediate AEP expression in PD patients’ gut (Fig. 7e). Indeed, both C/EBPβ and AEP are expressed in multiple cell types, including neurons. Recently, it has been reported that C/EBPβ in the microglia mediates Tau pathologies propagation in the AD brain^64^. Moreover, AEP has been shown to cleave Tau at N167 in microglia and mediates its uptake into microglia^65^. Conceivably, the crucial proteins may also facilitate a-Syn pathologies in other cell types in addition to TH-positive dopaminergic neurons.

A previous study shows that the progression of PD pathologies, including p-α-Syn, and α-Syn aggregation and dopaminergic neuronal loss, are reproduced by intragastric administration of low doses (5 mg/kg) of rotenone in mice. Rotenone is not detectable in the blood or the brain at such a dose or lower. The mice also display motor disorders 3 months after treatment^66,67^. Resection of vagus and sympathectomy nerve blocks rotenone-induced α-Syn aggregates spreading from the gut into the brain stem^44,68,69^. Inoculation of the insoluble fraction of PD brain into the duodenum of rat reveals α-Syn propagation into the brain stem of the animals^70^. Because PD patients display constipation prodromal symptoms but not stomach disorders, in our most recent study, we chose to inoculate preformed fibrils into the gut wall instead of the stomach, though the latter contains more vagus nerves than the former and revealed that colonoscopic injection of α-Syn N103/Tau N368 fibrils transport into the brain, resulting in PD-like pathologies and motor dysfunctions^45^. Thus, these studies strongly support that the vagus nerve mediates inter-synaptic cell-cell propagation and might mediates α-Syn spreading, and these findings are in alignment with a most recent report from other groups^68,69,71^. In the current work, we also found that chronic low dose of rotenone gastric exposure elicits Lewy body-like inclusion in the colon of α-SNCA mice, which propagates into the brain stem via the vagus nerve, for which the C/EBPβ/AEP axis is required (Fig. 6e). Conceivably, the reduction of α-Syn aggregates in the colon in α-SNCA/C/EBPβ^+/−^ and α-SNCA/AEP^−/−^ mice partially accounts for the diminished brain propagation of Lewy body inclusions. Moreover, it is also possible that C/EBPβ/AEP axis somehow mediates inter-synaptic transmission of the α-Syn fibrils or both of the mechanisms may contribute to α-Syn aggregates spreading from the ENS to the CNS in α-SNCA mice upon rotenone stimulation.

There are also some limitations to this study. Because the present study is a continuation of our previous results, hence, we did not include multiple control groups. Further, for the rare transgenic mice and human patient tissues, there is a lack of cross-verification for the Lewy body-like pathology with different α-Syn antibodies, although the pSer129 antibody is broadly employed in various reports, and we have qualified its specificity in the brain and gut tissues. In this study, we continually focus on the α-Syn pathology of TH-positive neurons. The results show that p-α-Syn S129 was co-stained with TH-positive cells in the colon tissues, indicating that p-α-Syn S129 was mainly aggregated in the TH-positive cells. Furthermore, to unveil the exact mechanism of the original a-Syn pathology in the gut, different cell markers in the colon will be explored in the future studies.

In the current study, we show that C/EBPβ/AEP pathway is age-dependently escalated in both the brain and gut in α-SNCA transgenic mice and human PD patients, correlating with Lewy body formation, inflammation, and oxidative stress augmentation. Depletion of them from α-SNCA mice diminishes α-Syn truncation by AEP, abolishing Lewy body formation. Remarkably, the temporal Lewy body spreading from the gut into the brain along the vagus nerve is abrogated, when C/EBPβ/AEP pathway is inactivated in α-SNCA mice. Hence, our study provides a molecular mechanism of how C/EBPβ/AEP signaling mediates Lewy body pathology propagation in PD pathogenesis during aging.

## Methods

### Animals

All mice were obtained from the Jackson Laboratory. Human SNCA overexpressing mice (B6.Cg-Tg(SNCA)OVX37Rwm Sncatm1Rosl/J, stock NO. 023837) without endogenous mouse α-syn. The AEP-knockout mice on a C57BL/6 background were generated as previously reported^25^. Since the homozygous mutation is lethal on pure strain backgrounds, *Cebpb* mice were maintained as heterozygotes on C57BL/6 strain backgrounds. α-SNCA mice were crossed with AEP^−/−^ mice to generate α-SNCA /AEP^−/−^ mice. α-SNCA mice were crossed with C/EBPβ^+/−^ mice to generate α-SNCA/C/EBPβ^+/−^ mice^72^. The following animal groups were analyzed: α-SNCA, α-SNCA /C/EBPβ^+/−^, α-SNCA /AEP^−/−^. Both female and male mice were used. The experimental groups were shown in the graphical scheme (Supplementary Fig. 1a). Animal care and handling were performed according to the Declaration of Helsinki and Emory Medical School guidelines. Investigators were blinded to the group allocation during the animal experiments. The protocol was reviewed and approved by the Emory Institutional Animal Care and Use Committee.

### Oral gavage

One-month-old α-SNCA, α-SNCA/AEP^−/−^ and α-SNCA/C/EBPβ^+/−^ mice were treated with 0.1 ml/25 grams of a vehicle containing 1% methylcellulose (Sigma Cat# M0512) and 1.25% chloroform (Sigma Cat#2432) or a solution containing 0.625 mg/ml of rotenone (ULTRA Scientific Cat#=PST-890) with 1% methylcellulose and 1.25% chloroform. We prepared rotenone solution by dissolving rotenone in chloroform and then diluting it into 1% methylcellulose solution while mixing vigorously. Rotenone or vehicle control treatment was orally administered with a 1.2 × 60 mm gavage (Unimed, Switzerland) once a day for 5 days/week, consecutively for 3 weeks, 6 weeks, or 12 weeks separately.

### Antibodies and reagents

Antibodies to the following targets were used: Antibody to C/EBPβ (Santa Cruz Biotechnology, sc-7962), AEP antibody clone 6E3 and 11B7 (from Dr. Colin Watts, University of Dundee), AEP (Cell Signaling Technology, 93627 S), NQO1 (Cell Signaling Technology, 3187 s, and Santa Cruz Biotechnology, sc-32793), TH (Cell Signaling Technology, 2792 s), MAO-B (GeneTex, GTX113771), α-Synuclein (Santa Cruz Biotechnology, sc-69977), α-Syn303 (Biolegend, MMS-5085), p-α-syn S129 (Santa Cruz Biotechnology, sc-135638), α-syn N103 (Ye Lab, its specificity was validated in our previous publication^31^) and β-actin (Sigma-Aldrich, A5316), Ub (Santa Cruz, SC-8017), IBA1 (VWR, 019-19741). All chemicals not included above were purchased

### Human tissue samples

Postmortem brain freeze samples and paraffin-embedded sections of four PD patients (age 71.3 ± 10.8 years, mean ± SD) and four non-PD controls (age 71 ± 10.1 years) were provided by the Emory Alzheimer’s Disease Research Center. The details are presented in Table S2, Supporting Information. PD cases were clinically diagnosed and neuropathologically confirmed, and all the PD patients are idiopathic PD (iPD), the same as our previous study^73,74^. The SN area was selected according to the human brain anatomical map and further confirmed by TH staining. The SN part of frozen samples was used for the western blotting. PD patients’ gut biopsies were the same as in our previous study^73^. The included subjects were recruited from patients (55–70 years old) suffering from PD and constipation (*n* = 4). PD was diagnosed according to MDS clinical diagnostic criteria. Constipation was assessed using Patient Assessment of Constipation symptom (PAC-SYM). The excluded subjects were those with Parkinson’s syndrome of non-iPD, colon disease, and other digestive diseases. The age-matched subjects without PD and constipation were selected as the control group (*n* = 4). Endoscopic biopsies were performed on all subjects. The colon tissue specimens of 0.8 mm thickness were collected about 5 cm away from the ileocecal valve. The study was approved by the biospecimen committee at Emory University. PD cases were clinically diagnosed and neuropathologically confirmed. Informed consent was obtained from all cases.

### Western blot analysis

The mouse brain or human tissues were lysed in lysis buffer (50 mM Tris, pH 7.4, 40 mM NaCl, 1 mM EDTA, 0.5% Triton X-100, 1.5 mM Na3VO4, 50 mM NaF, 10 mM sodium pyrophosphate, 10 mM sodium β-glycerophosphate, supplemented with a cocktail of protease inhibitors), and centrifuged for 15 min at 16,000 × *g*. The supernatant was boiled in an SDS loading buffer. After SDS-PAGE, the samples were transferred to a nitrocellulose membrane. The membrane was blocked with TBS containing 5% nonfat milk and 0.1% Tween 20 (TBST) at RT for 1 h, followed by the incubation with the primary antibody at 4 °C overnight, and with the secondary antibody at RT for 1 h. The samples for analyzing the level of soluble and insoluble α-synuclein were prepared in our previous study (Chen et al. Adv Sci. 2022). The supernatants from Triton X-100 cell lysates were collected as soluble fractions. The resultant pellets were further lysed in the solution containing 2% SDS and 8 m urea, sonicated at 30 W for 1 s ten times, and centrifuged at 100,000 × *g* for 30 min. Resultant supernatants were collected as insoluble fractions. The supernatants were boiled in 1× SDS loading buffer. After SDS-PAGE, the samples were transferred to a nitrocellulose membrane. Western blot analysis was carried out with the antibodies. Primary antibodies to the following targets were used: C/EBPβ (1:1000), AEP (6E3, 1:1000), AEP (1:1000), NQO1 (1:1000), TH (1:1000), MAO-B (1:1000), MPO (1:1000), α-syn (1:1000), p-α-syn S129 (1:1000), N103 (1:1000), and beta-actin (1:5000). After washing with TBST, the membrane was developed using the enhanced chemiluminescent (ECL) detection system. All blots or gels derive from the same experiment, and they were processed in parallel. Quantifications were performed by analyzing the relative densities of the exposed film using ImageJ, and the relative ratio of the band density was used for the statistics.

### Immunostaining

The immunostainings were performed on 30-μm frozen sections of mouse tissue and 5-μm paraffin-embedded human tissue sections. For immunohistochemical (IHC) staining, the sections were treated with 0.3% H_2_O_2_ for 10 min. Sections were washed three times in PBS and blocked in 1% BSA, 0.3% Triton X-100, for 30 min, followed by overnight incubation with C/EBPβ (1:300), AEP (11B7, 1:500), IBA1 (1:500), TH (1:500), p-a-Synuclein 129 (1:300), and N103 (1: 500) at 4 °C. The signal was developed using the Histostain-SP kit (Invitrogen, #95-6143). For immunofluorescence, the sections were incubated overnight with various primary antibodies of TH (1:500), AEP (11B7, 1:500), C/EBPβ (1:300), N103 (1: 500), 4-HNE (1: 50), Ub (1:300), and IBA1 (1:500) at 4 °C. Then, the sections were incubated with the matched fluoro-conjugated secondary antibody for 2 h at room temperature, after three times washing them in PBS. The slides were washed three times in PBS and covered with glass using the mounting solution, after DAPI staining for 5 min. The immunoreactivity in the section was quantified using fluorescence intensity with ImageJ software and the intensity of each to the control group as the fold. For each animal, the same level of three consecutive sections of the SN and striatum and gut tissues were analyzed. The values of fluorescence intensity were calculated on average from each group. The conditions of the analysis were blinded to the investigator.

### AEP activity assay

Assay buffer (20 mM citric acid, 60 mM Na2HPO4, 1 mM EDTA, 0.1% CHAPS, and 1 mM DTT, pH 6.0) of 100 μl including 100 μM AEP substrate Z-Ala-Ala-Asn-AMC (ABchem) was added to tissue lysates. AMC released by substrate cleavage was quantified using a fluorescence plate reader at 460 nm for 1 h in kinetic mode.

### MAO-B activity assay

Tissue lysates (20 μg) were incubated with the working solution of 100 μl containing 400 μM Amplex Red reagent, 2 U/ml HRP, and 2 mM benzylamine substrate (Molecular Probes). The fluorescence of MAO-B activity was measured in a fluorescence plate reader using excitation in the range of 530–560 nm and emission at 590 ± 10 nm at 37 °C for 2 h in kinetic mode.

### ROS detection

Chloromethyl-H2DCFDA (CM-H2DCFDA) was used as a probe for the assay of reactive oxygen species content. Fresh tissue lysates in pre-warmed PBS buffer containing the probe to provide a final working concentration of 5 μM at 37 °C for 30 min. The reaction was terminated by chilling the reaction mixture in ice. The formation of the oxidized fluorescent derivative (DCF) was monitored at EX/EM of 485/528 nm using a fluorescence spectrophotometer. All procedures were performed in the dark 96-well plates with blanks containing only CM-H2DCFDA as the measurement of autofluorescence.

### ELISA quantification of inflammatory cytokine

For the quantification of inflammatory cytokine, tissue lysates were analyzed with mouse IL-6 (Thermo Fisher BMS603HS), IL-1β (Thermo Fisher 88-5019-22), and TNF-α (Thermo Fisher BMS607-3) ELISA kits according to the manufacturer’s instructions. The sample concentrations were determined by comparison with the standard curve.

### Behavioral test

The behavioral test included the rotarod test and the grid test. For the rotarod test, animals were trained for 2 min at a speed of 4 rpm. After this initial training, mice performed eight trials for a maximum of 5 min with increasing speed starting from 4 rpm and increasing to 40 rpm. The fall-off time was recorded. For the grid test, mice were placed in the center of a 30 cm × 30 cm screen with a 1 cm wide mesh. The screen was inverted head-over-tail and placed on supports 40 cm above an open cage with deep bedding. Mice were timed until they released their grip or remained for 60 s.

### Gastrointestinal motility assays

Stool water content was measured in mice as described previously^75^. Red carmine dye test was used to measure the total GI tract transit time^76^.

### Stereological quantification and statistical analysis

The number of TH-positive cells in the SN was estimated with a random-sampling stereological counting method. For each animal, every sixth section (30-μm) throughout the rostrocaudal extent of the SN and every sixth section covering the entire extent of the STR were incorporated into the counting procedure. The investigator was blinded to the conditions of the experiment. They were analyzed under the randomly placed counting frames (50 μm × 50 μm) on a counting grid (120 μm × 120 μm). An optical dissector of 22 μm with 2 μm upper and lower guard zones was used. The SN boundaries were outlined under magnification of the 5× objective lens and cells were counted with the 40× objective lenses using an Olympus BX53 microscope. The total number of neurons in the SN was estimated using the optical fractionator method. For quantification of positive cells, stained color was selected and the proper threshold was set for the binarization of the selected color image. The total number of immunoreactive neurons was analyzed using the same threshold (Image J). The conditions of the analysis were blinded to the investigator. Statistical analysis was performed using either Student’s *t*-test (two-group comparison) or one-way ANOVA followed by LSD post hoc test (more than two groups), and differences with *P* < 0.05 were considered significant.

### Reporting summary

Further information on research design is available in the Nature Research Reporting Summary linked to this article.

## Supplementary information

Supplemental material

Supplemental material uncropped blots

Reporting Summary

## Data Availability

The data that support the findings of this study does not present restrictions and are available upon request for information that is made and that can be provided by the corresponding author.
