# Supplementary material for: C/EBPβ/AEP is age-dependently activated in Parkinson’s disease and mediates α-synuclein in the gut and brain
Source: NPJ Parkinsons Dis. 2023 Jan 6;9:1. doi: 10.1038/s41531-022-00430-8 (PMC9822984; doi:10.1038/s41531-022-00430-8)
Supplement: Supplementary file 1 — Supplemental material [file 41531_2022_430_MOESM1_ESM.pdf]

Supplementary Figure 1

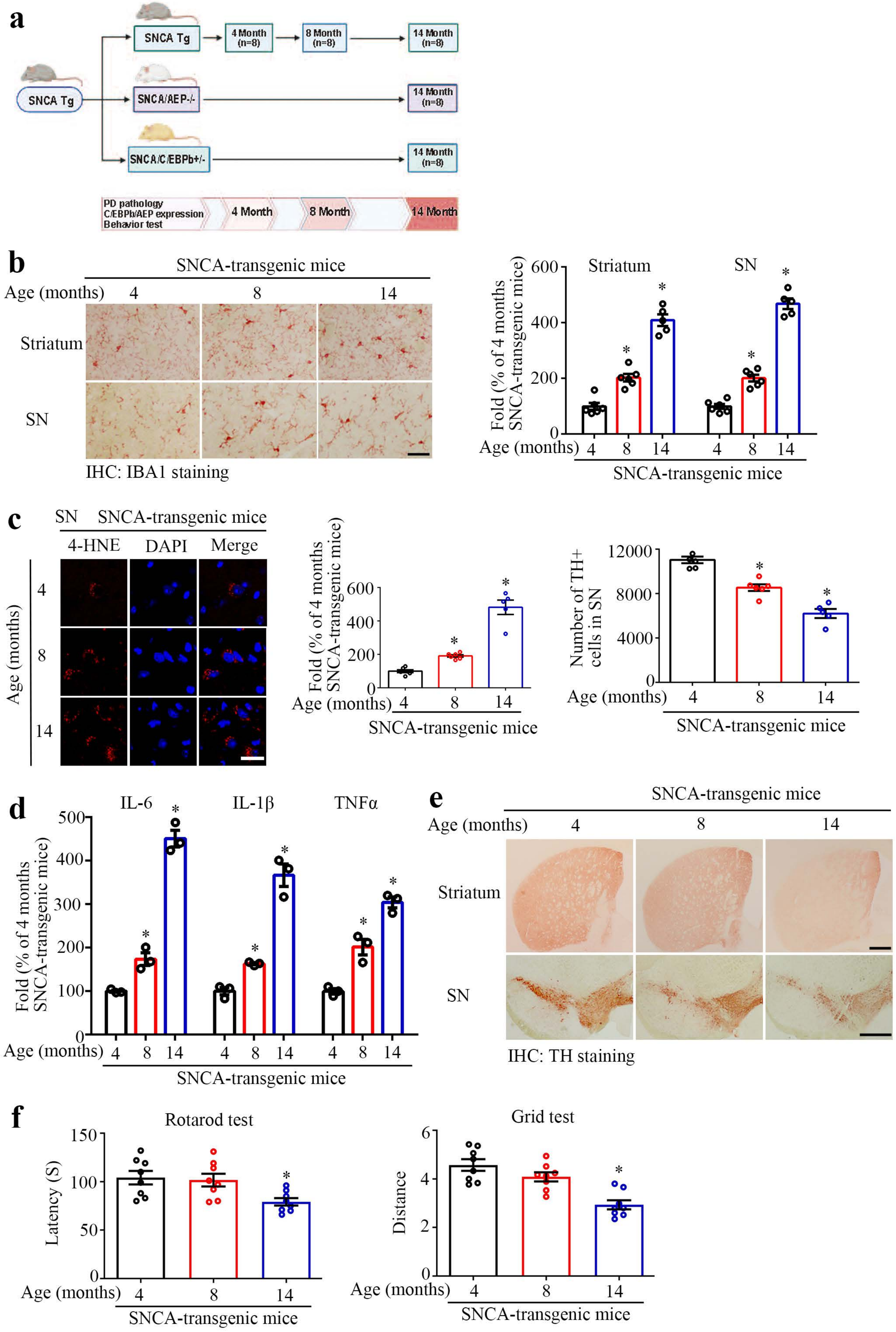

Supplementary figure 1. Microglia activation and oxidative stress are age-dependently elevated in α-SNCA mice.

(a) The graphical scheme of the experimental groups. (b) Immunostaining and the quantification of Iba1 in the striatum and SN. (n = 5-6 per group, *$P < 0.05$ *vs* 4 months old α-SNCA transgenic mouse, one-way ANOVA). Scale bar, 50 μm. (c) 4-HNE staining and the quantification in the SN. (n = 5-6 per group, *$P < 0.05$ *vs* 4 months old α-SNCA transgenic mouse, one-way ANOVA). Scale bar, 20 μm. (d) The expression levels of inflammatory cytokines in α-SNCA transgenic mice during aging. (n = 3 per group, *$P < 0.05$ *vs* 4 months old α-SNCA transgenic mouse, one-way ANOVA). (e) Immunostaining of TH in the striatum and SN, and the quantification of TH in SN. Scale bar, 50 μm. (n = 5-6 per group, *$P < 0.05$ *vs* 4 months old α-SNCA transgenic mouse, one-way ANOVA). (f) Rotarod test and Grid test. (mean ± s.e.m.; n = 8 mice per group; *$P < 0.05$ *vs* 4 months old α-SNCA transgenic mouse, one-way ANOVA).

Supplementary Figure 2

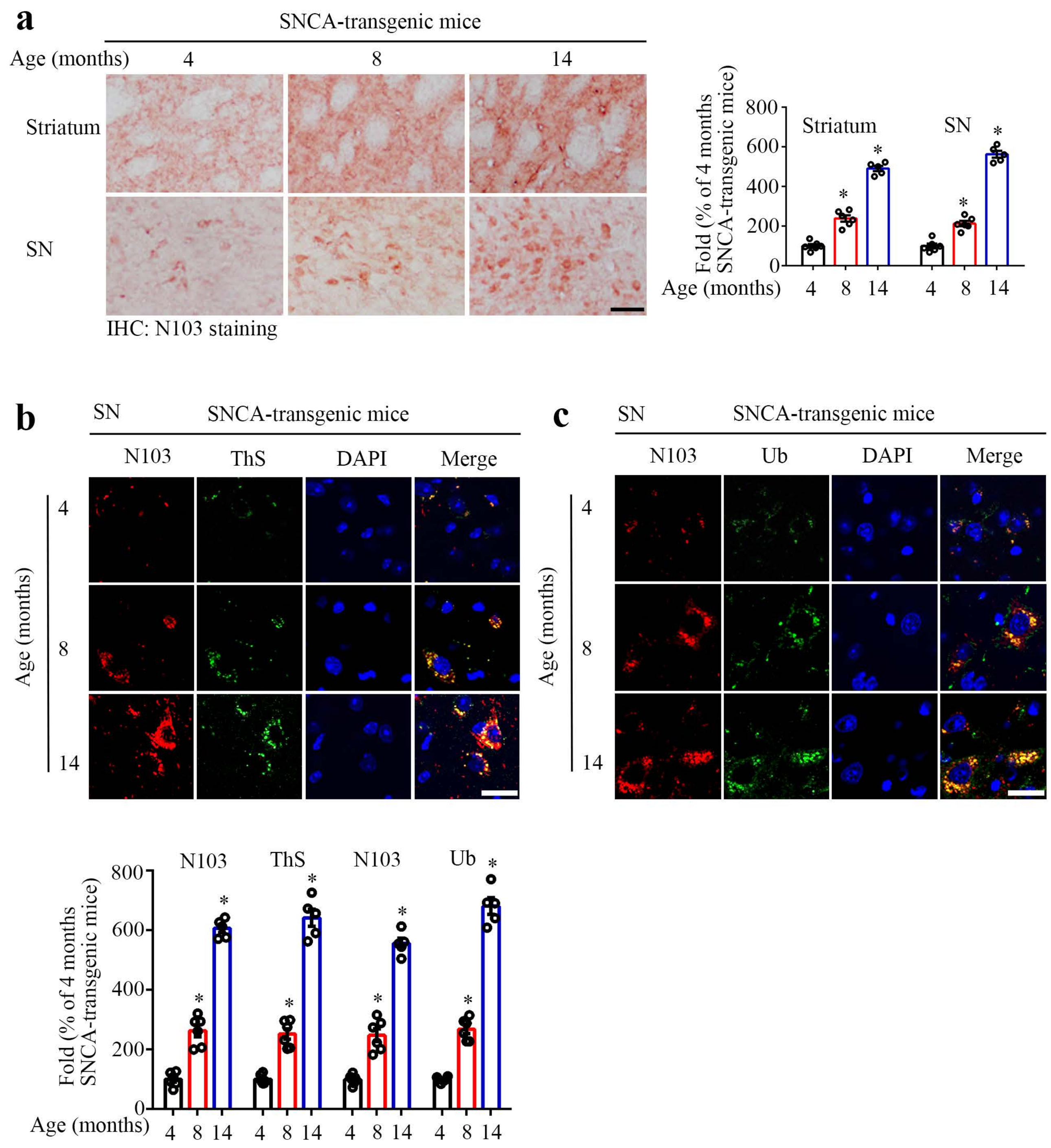

Supplementary figure 2. α-Syn is proteolytically cleaved at N103 and aggregated in an age-dependent manner in human α-SNCA mice. (a) Immunohistochemistry and the quantification of the α-Syn N103 in the striatum and SN of the α-SNCA transgenic mouse during aging. (n = 5-6 per group, *$P < 0.05$ *vs* 4 months old α-SNCA transgenic mouse, one-way ANOVA). Scale bar, 50 μm. Immunofluorescent co-staining and the quantification of α-Syn N103 and ThS (b), α-Syn N103 and Ub (c) in SN. (n = 5-6 per group, *$P < 0.05$ *vs* 4 months old α-SNCA transgenic mouse, one-way ANOVA). Scale bar, 20 μm.

Supplementary Figure 3

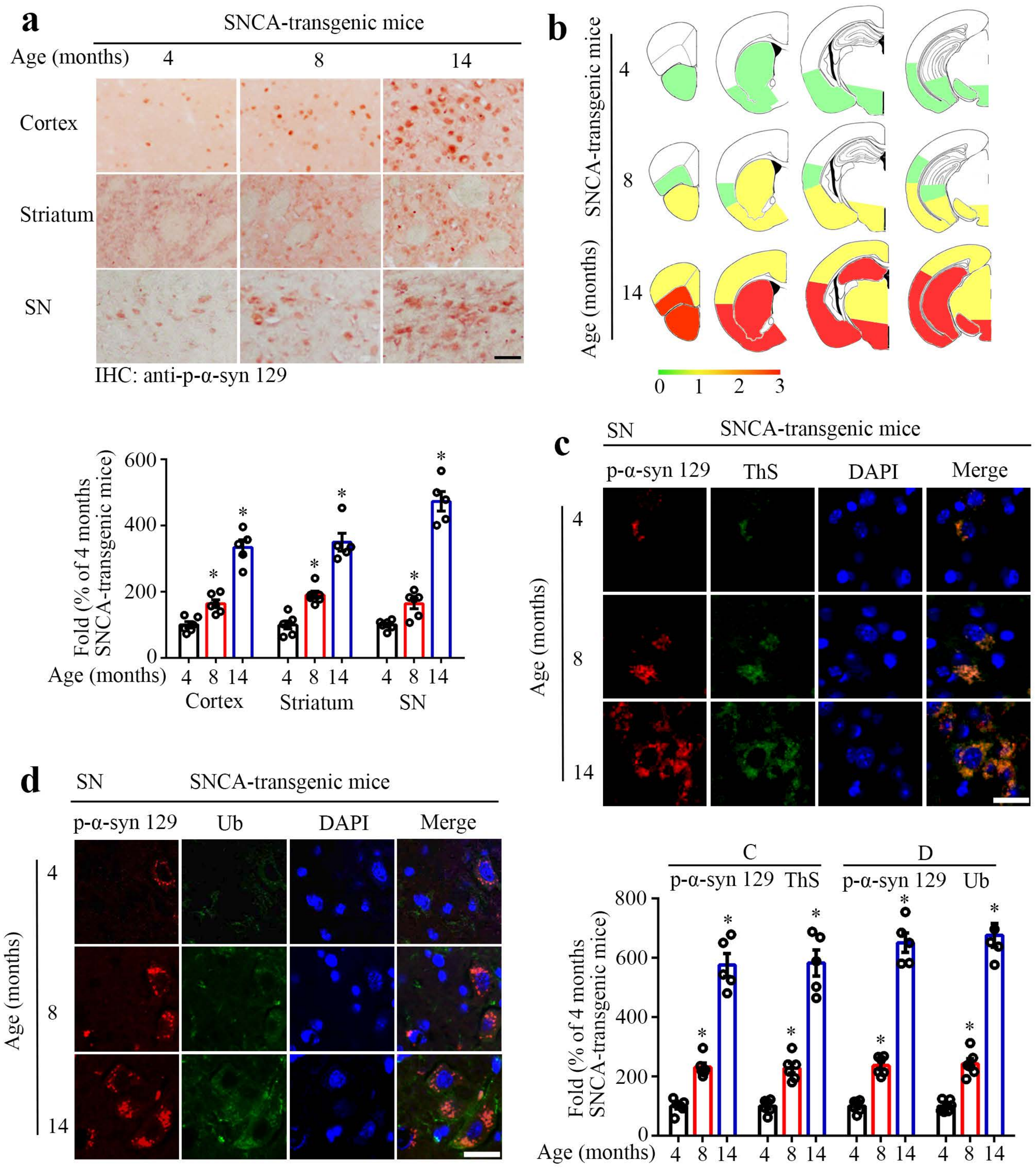

Supplementary figure 3. α-Syn S129 phosphorylation and Lewy body formation are escalated in an age-dependent way in α-SNCA mice. (a) Immunohistochemistry and the quantification of p-α-Syn 129 in the cortex, striatum, and SN. (n = 5-6 per group, $*P < 0.05$ *vs* 4 months old α-SNCA transgenic mouse, one-way ANOVA). Scale bar, 50 μm. (b) Heat map colors represent the extent of p-α-Syn 129 pathologies (white (0), no pathology; red (3), the maximal pathology). Immunofluorescent co-staining and the quantification of p-α-Syn 129 and ThS (c), p-α-Syn 129, and Ub (d) in SN. (n = 5-6 per group, $*P < 0.05$ *vs* 4 months old α-SNCA transgenic mouse, one-way ANOVA). Scale bar, 20 μm.

Supplementary Figure 4

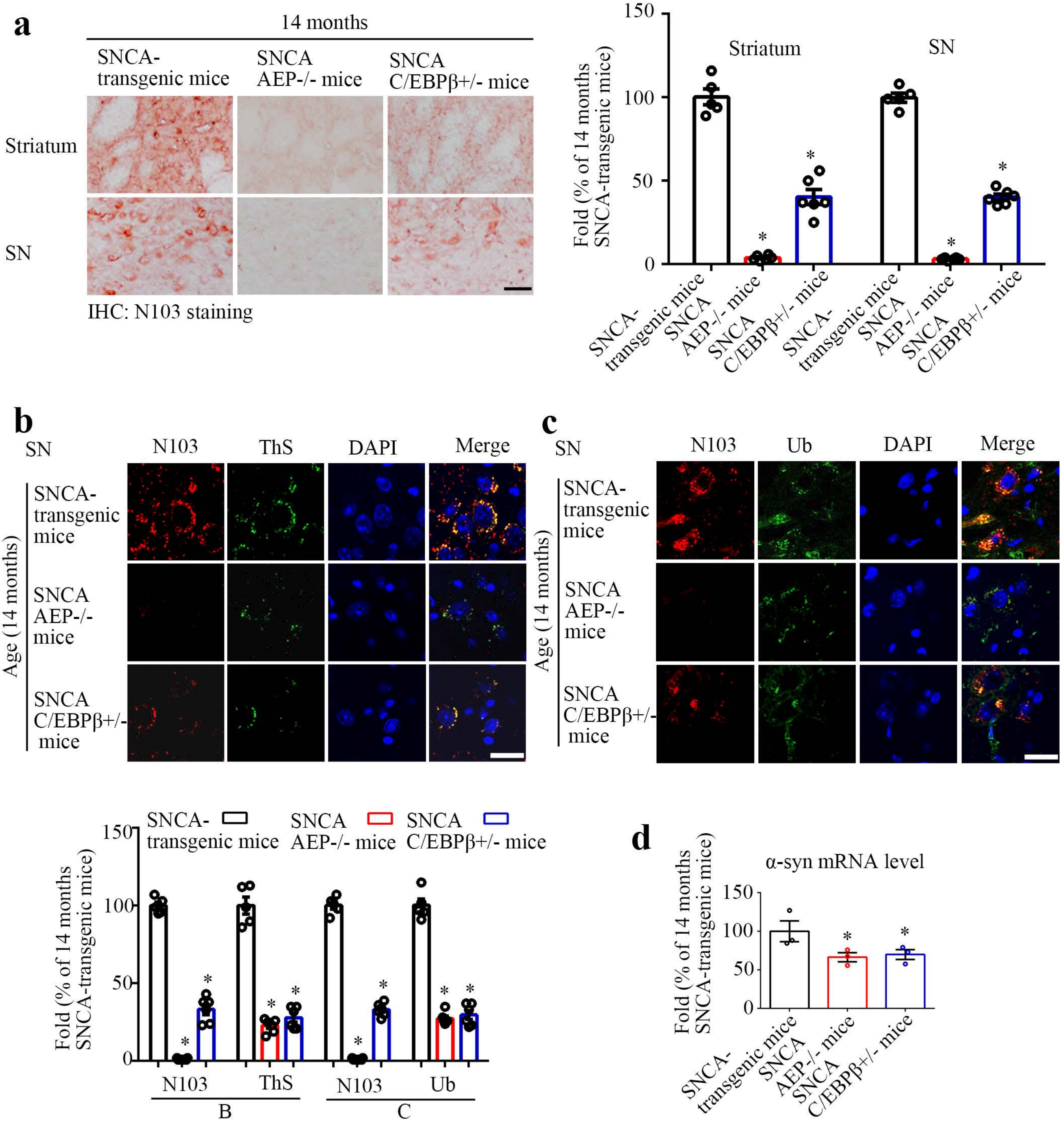

Supplementary figure 4. α-Syn N103 fragmentation and aggregation are attenuated when C/EBPβ or AEP is depleted in human α-SNCA mice. (a) Immunohistochemistry and the quantification of the α-Syn N103 in the striatum and SN. (n = 5-6 per group, $^*P < 0.05$ *vs* 14 months old α-SNCA transgenic mouse, one-way ANOVA). Scale bar, 50 μm. Immunofluorescent co-staining and the quantification of α-Syn N103 and ThS (b), α-Syn N103 and Ub (c) in SN. (n = 5-6 per group, $^*P < 0.05$ *vs* 14 months old α-SNCA transgenic mouse, one-way ANOVA). Scale bar, 20 μm.

Supplementary Figure 5

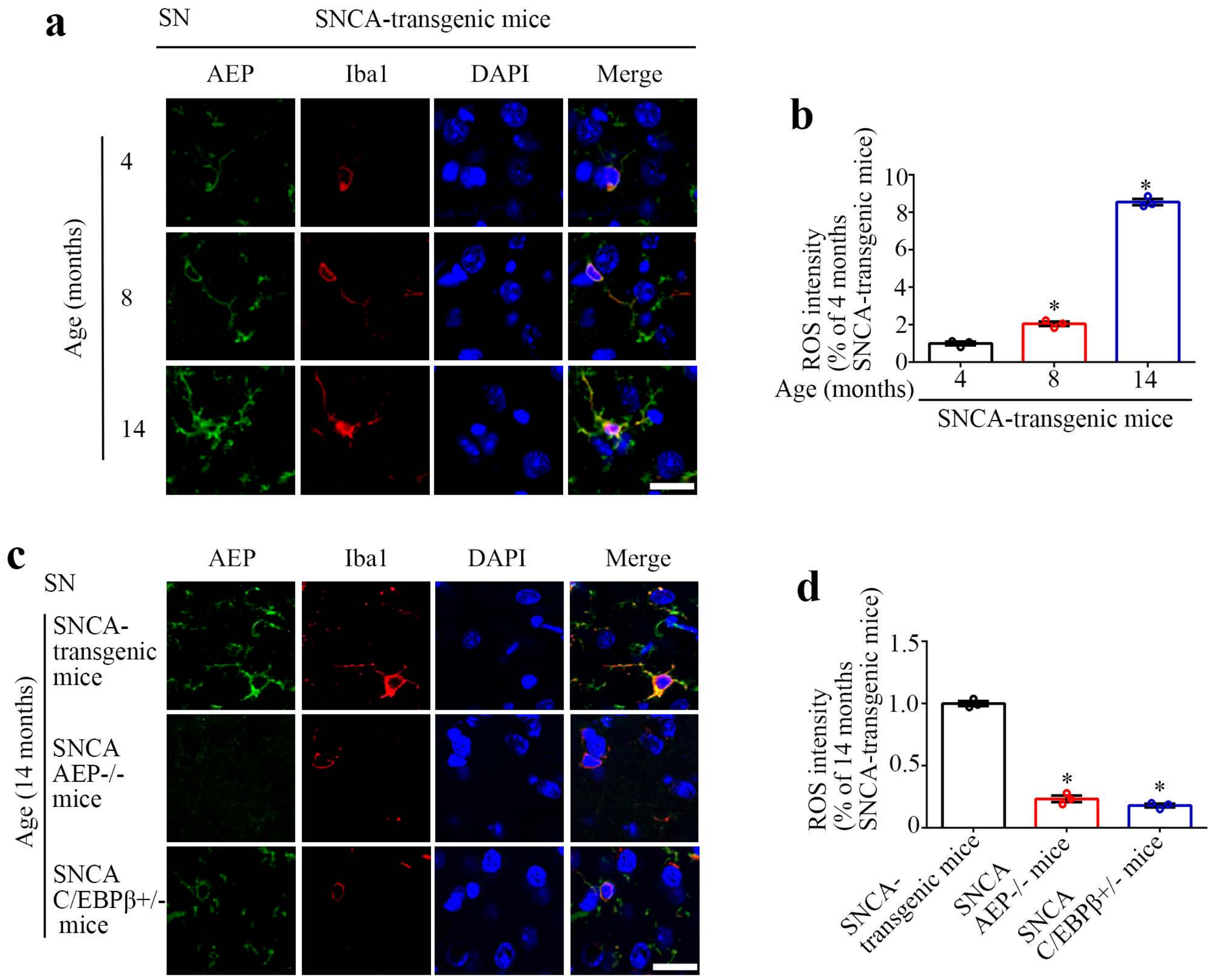

Supplementary figure 5. C/EBPβ/AEP axis mediates microglia activation and ROS in human α-SNCA mice. (a) Immunofluorescent co-staining of AEP and Iba1 and ROS intensity (b) in SN of the α-SNCA transgenic mouse during aging. Scale bar, 20 μm. (n = 3 per group, **P* < 0.05 *vs* 4 months old α-SNCA transgenic mouse, one-way ANOVA). (c) Reduction of C/EBPβ and knockdown of delta-secretase decreases the expression of AEP, Iba1, and ROS intensity (d) in the α-SNCA transgenic mouse. Scale bar, 20 μm. (n = 3 per group, **P* < 0.05 *vs* 14 months old α-SNCA transgenic mouse, one-way ANOVA).

Supplementary Figure 6

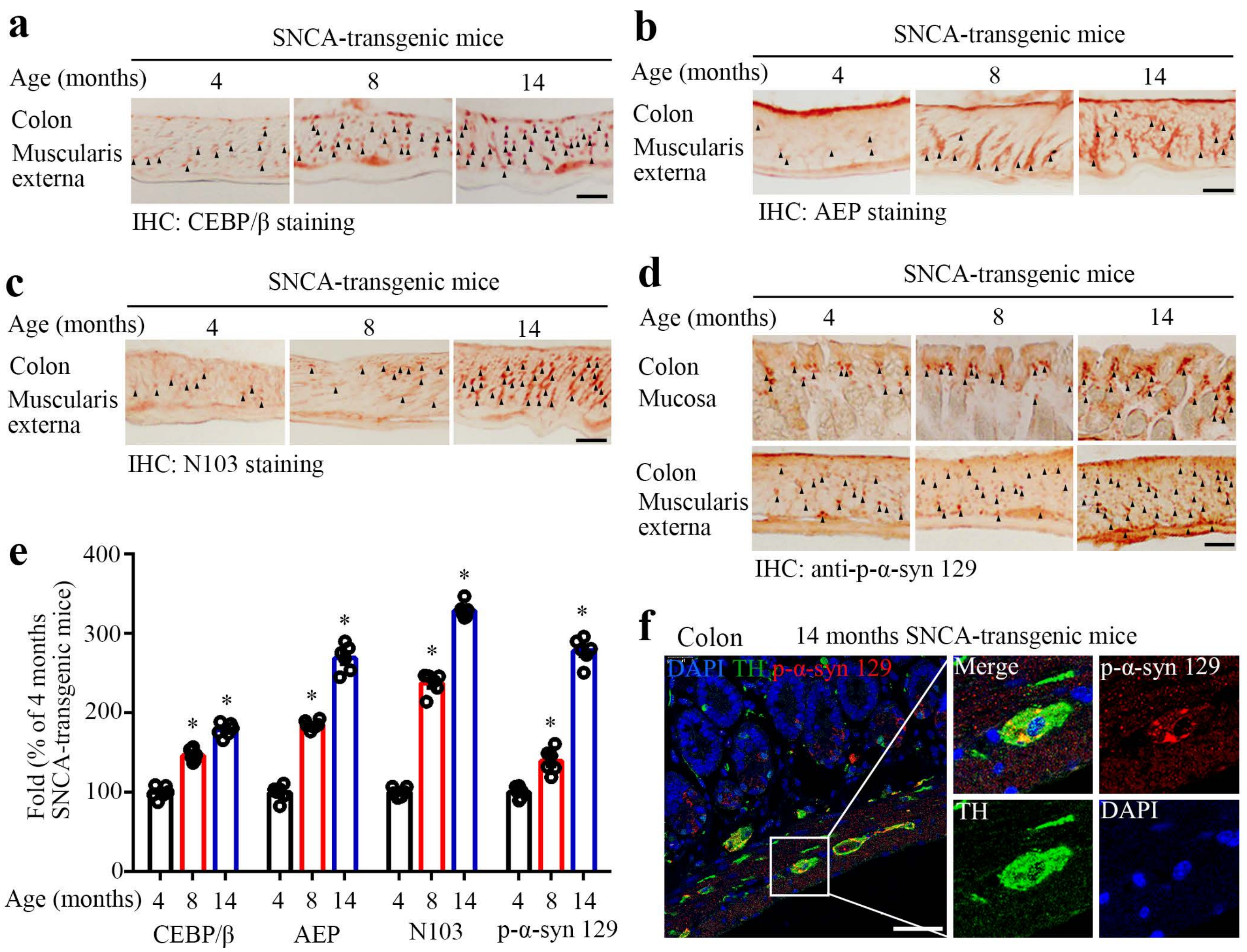

Supplementary figure 6. C/EBPβ/AEP axis activation and Lewy body-like pathology in the gut of α-SNCA mice. Immunohistochemistry of the C/EBPβ (a), AEP (b), α-Syn N103 (c), p-α-Syn 129 (d), and the quantification (e) in the colon of α-SNCA transgenic mouse during aging. The co-staining of TH and p-α-Syn 129. Scale bar, 50 μm. (the positive signals were indicated with a black triangle, n = 6 per group, *$P < 0.05$ *vs* 4 months old α-SNCA transgenic mouse, one-way ANOVA).

Supplementary Figure 7

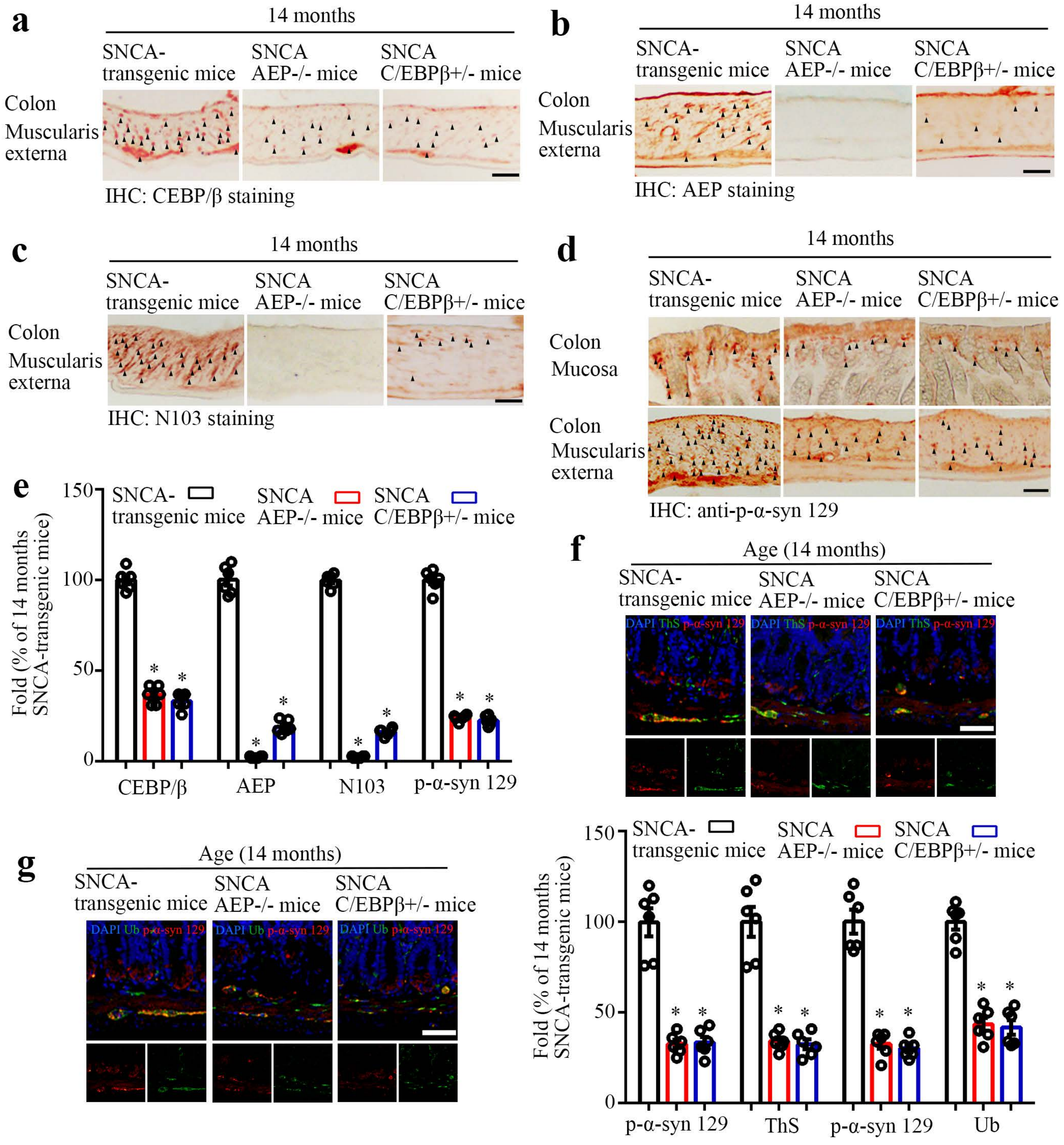

Supplementary figure 7. Inactivation of C/EBPβ/AEP in α-SNCA mice blunts Lewy body-like pathology in the gut. Reduction of C/EBPβ and knockdown of delta-secretase decreases the expression of the C/EBPβ (a), AEP (b), α-Syn N103 (c), p-α-Syn 129 (d), and the quantification (e) in the colon of α-SNCA transgenic mouse. Scale bar, 50 μm. (the positive signals were indicated with a black triangle, n = 6 per group, $*P < 0.05$ *vs* 14 months old α-SNCA transgenic mouse, one-way ANOVA). Immunofluorescent co-staining and the quantification of p-α-Syn 129 and ThS (f), p-α-Syn 129, and Ub (g) in the colon. (n = 6 per group, $*P < 0.05$ *vs* 14 months old α-SNCA transgenic mouse, one-way ANOVA). Scale bar, 50 μm.

Supplementary Figure 8

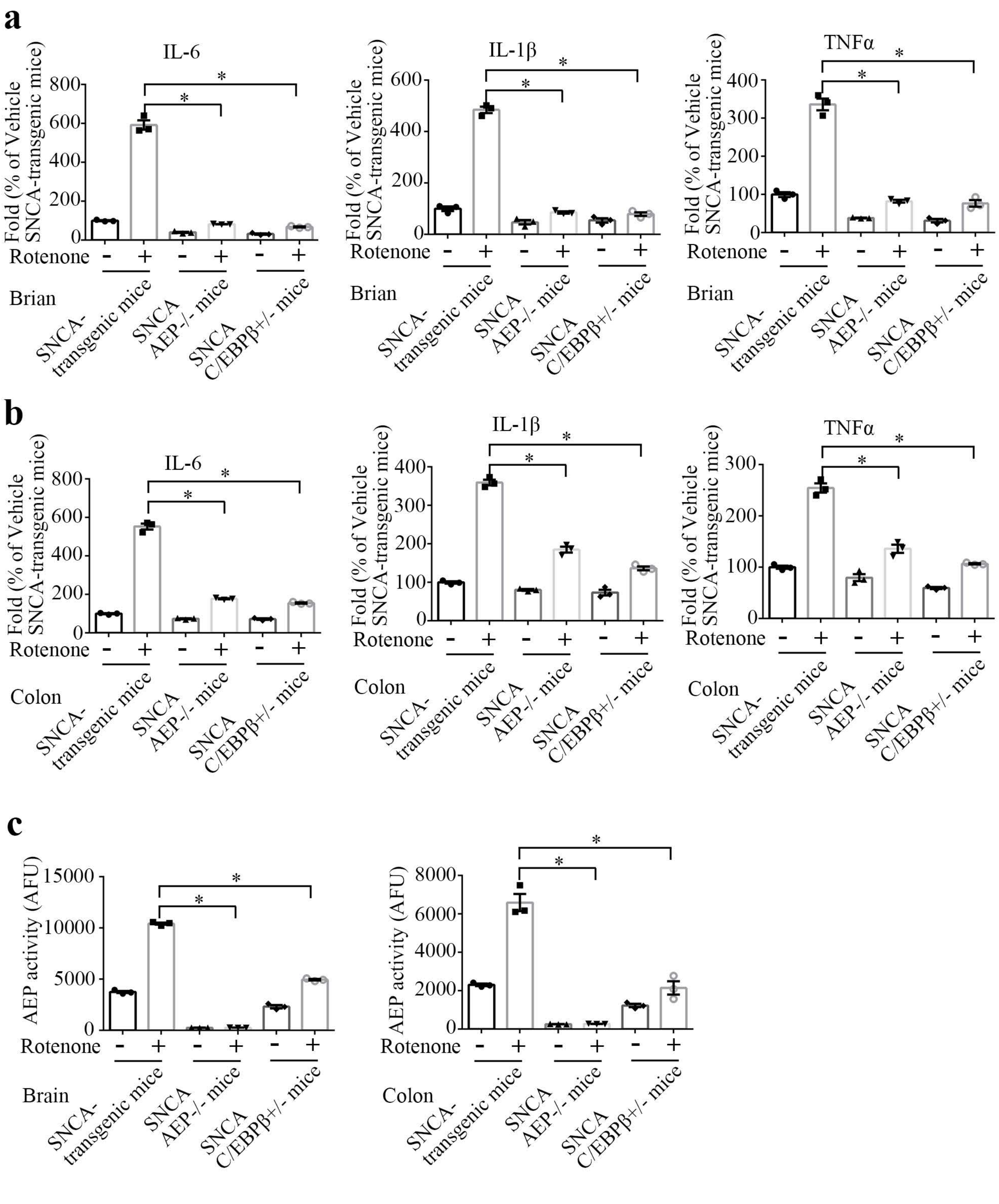

Supplementary figure 8. C/EBPβ/AEP knockout mitigates inflammation in the gut and brain of α-SNCA mice. Reduction of C/EBPβ and knockdown of delta-secretase decreases the expression levels of inflammatory cytokines in the brain (a) and colon (b) of the α-SNCA transgenic mouse after rotenone treatment. (n = 3 per group, **P* < 0.05 *vs* α-SNCA transgenic mouse, one-way ANOVA). (c) AEP activity assay in the brain and colon samples (n = 3 per group, **P* < 0.05 *vs* α-SNCA transgenic mouse, one-way ANOVA).

Supplementary Table. Human Samples Information

**Cases**	**Age at Onset**	**Age at Death**	**Sex**
Control	X	68	male
Control	X	85	male
Control	X	61	female
Control	X	70	male
PD	74	84	female
PD	67	74	male
PD	52	58	male
PD	48	69	male
