# Supplementary figures and images for: C/EBPβ/AEP is age-dependently activated in Parkinson’s disease and mediates α-synuclein in the gut and brain
Source: NPJ Parkinsons Dis. 2023 Jan 6;9:1. doi: 10.1038/s41531-022-00430-8 (PMC9822984; doi:10.1038/s41531-022-00430-8)

Figure 1

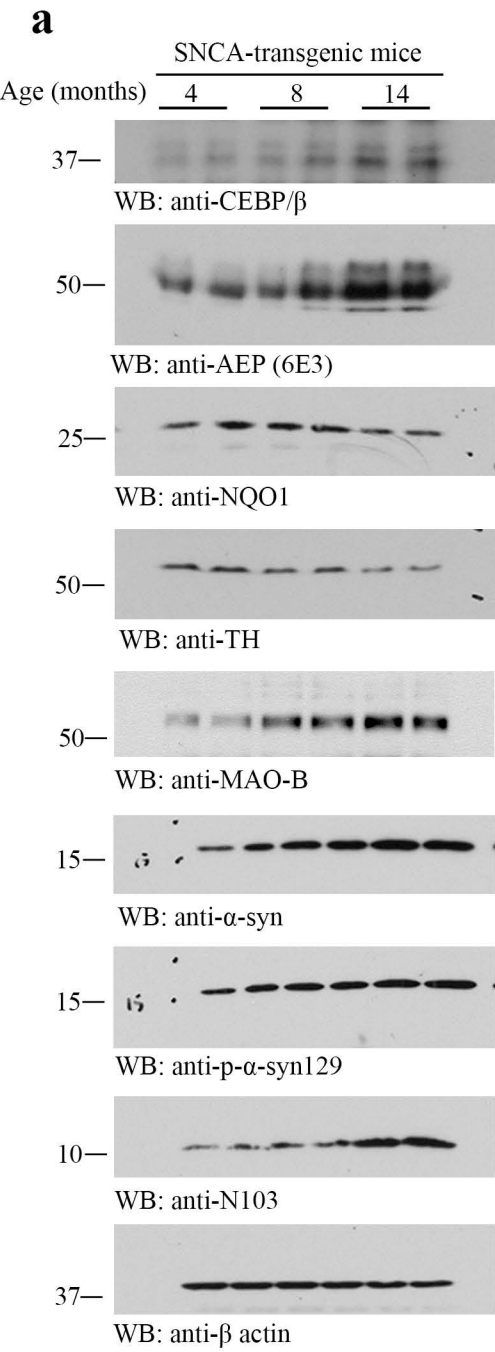

Figure 2

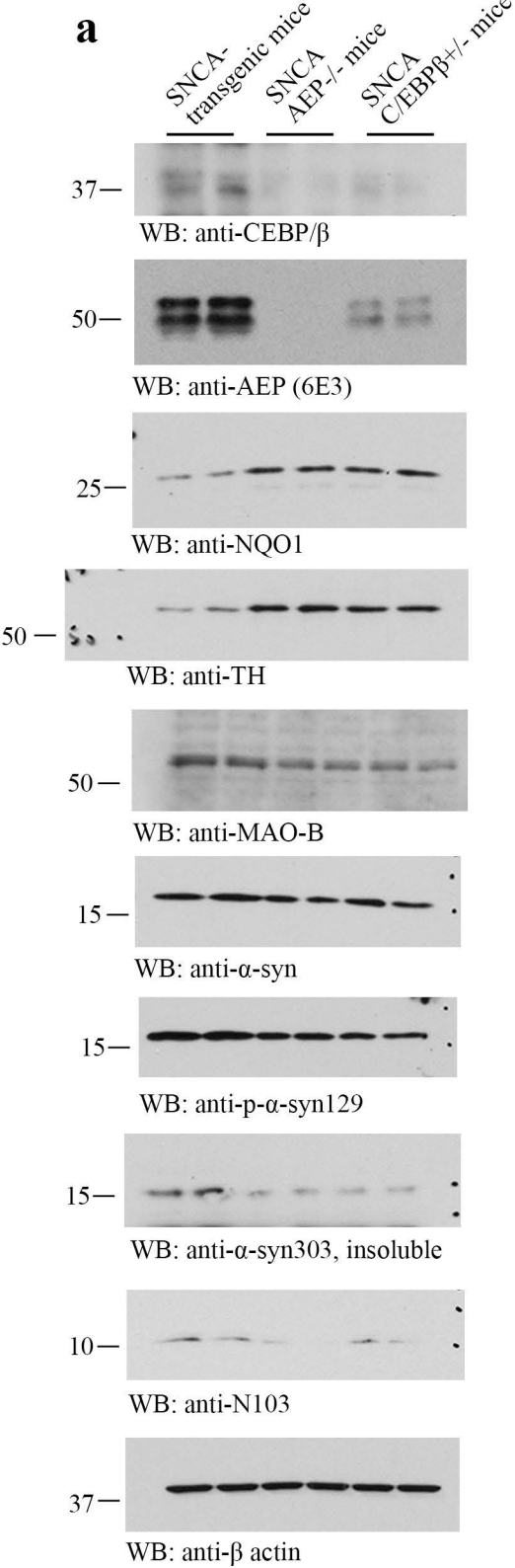

Figure 5

**b**

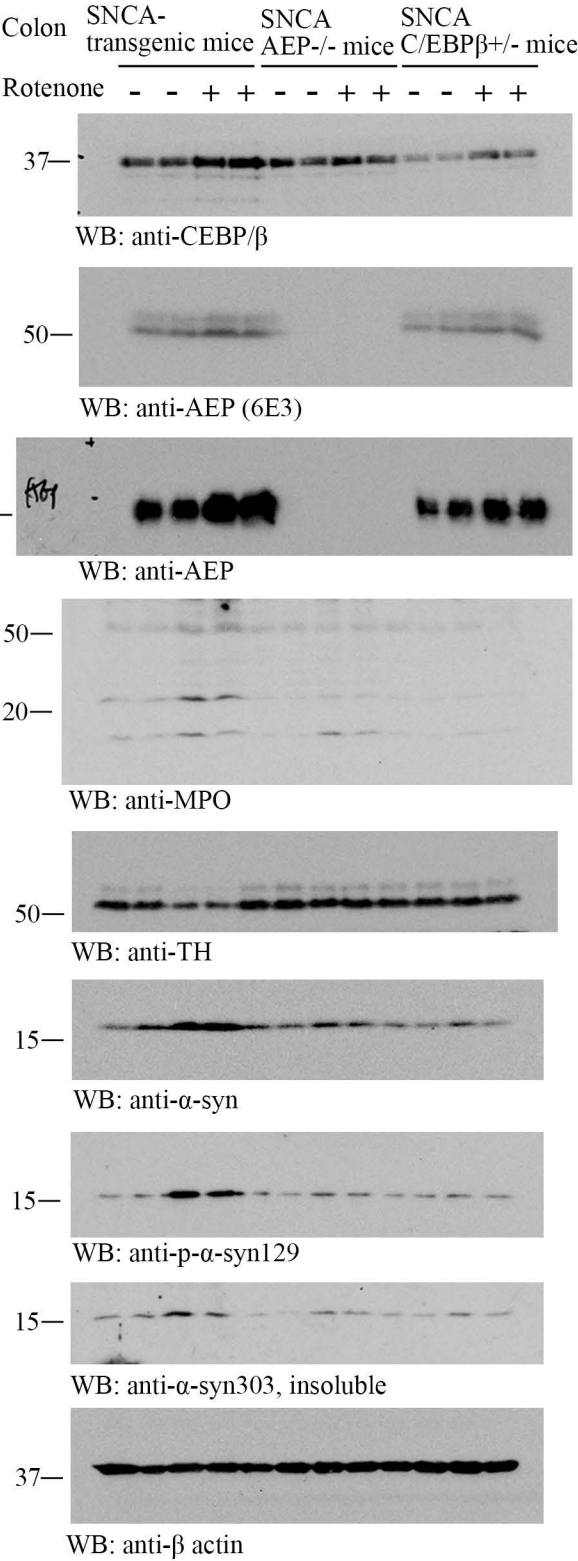

**c**

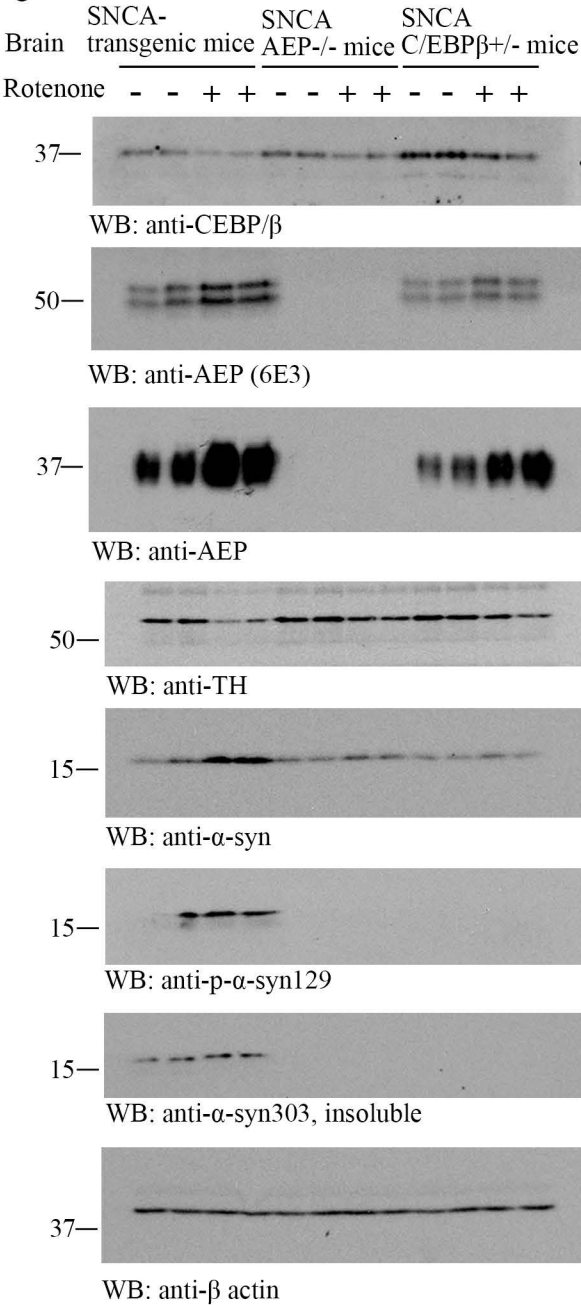

Figure 7

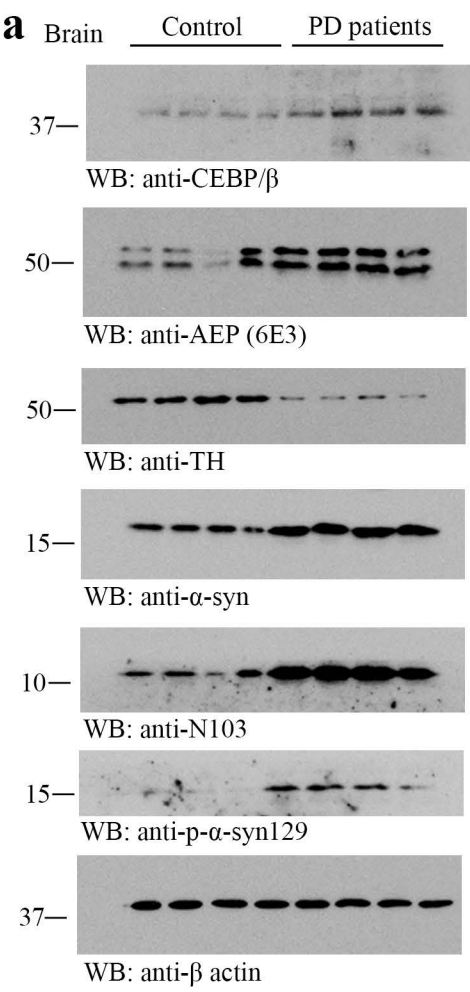

Supplement: Supplementary file 2 — Supplemental material uncropped blots [file 41531_2022_430_MOESM2_ESM.pdf]
